# A four-compartment controller model of muscle fatigue for static and dynamic tasks

**DOI:** 10.3389/fphys.2025.1518847

**Published:** 2025-02-12

**Authors:** James Yang, Ritwik Rakshit, Shuvrodeb Barman, Yujiang Xiang

**Affiliations:** ^1^ Human-Centric Design Research Lab, Department of Mechanical Engineering, Texas Tech University, Lubbock, TX, United States; ^2^ School of Mechanical and Aerospace Engineering, Oklahoma State University, Stillwater, OK, United States

**Keywords:** muscle fatigue model, isometric tasks, isokinetic tasks, compartment theory, central fatigue, peripheral fatigue

## Abstract

**Introduction:**

Compartment based models of muscle fatigue have been particularly successful in accurately modeling isometric (static) tasks or actions. However, dynamic actions, which make up most everyday movements, are governed by different central and peripheral processes, and must therefore be modeled in a manner accounting for the differences in the responsible mechanisms. In the literature, a three-component controller (3CC) muscle fatigue model (MFM) has been proposed and validated for static tasks. A recent study reported a four-compartment muscle fatigue model considering both short- and long-term fatigued states. However, neither has been validated for both static and dynamic tasks.

**Methods:**

In this work we proposed a new four-compartment controller model of muscle fatigue with enhanced recovery (4CCr) that allows the modeling of central and peripheral fatigue separately and estimates strength decline for static and dynamic tasks. Joint velocity was used as an indicator of the degree of contribution of either mechanism. Model parameters were estimated from part of the experimental data and finally, the model was validated through the rest of experimental data that were not used for parameter estimation.

**Results:**

The 3CC model cannot capture the fatigue phenomenon that the velocity of contraction would affect isometric strength measurements as shown in experimental data. The new 4CCr model maintains the predictions of the extensively validated 3CC model for static tasks but provides divergent predictions for isokinetic activities (increasing fatigue with increasing velocity) in line with experimentally observed trends. This new 4CCr model can be extended to various domains such as individual muscle fatigue, motor units’ fatigue, and joint-based fatigue.

## 1 Introduction

Localized muscular fatigue (LMF) is an exercise-induced reduction in the ability of a muscle to generate force or power (Bigland-Ritchie and Woods, 1984) and is an important consideration in obtaining accurate estimates of strength. While LMF is complex in its origins, all causative mechanisms can be classified as either central or peripheral. Central mechanisms originate in the central nervous system (CNS) and result in the impaired voluntary activation of motor units (MU), while peripheral mechanisms involve any and all effects distal to the neuromuscular joint, including muscle action potential propagation, excitation-contraction coupling (ECC), and chemical changes within the contractile elements in muscle. Numerous studies have investigated the relation between muscle shortening velocity, strength decline, and the mechanism(s) responsible with varied results.

### 1.1 Velocity influences strength

Increasing peak torque loss with increasing joint velocity has previously been observed (Bogdanis et al., 2007; Mathiassen, 1989; Matsuura et al., 2011; Morel et al., 2015; Perry-Rana et al., 2002), although Dalton et al. (2012) reported greater decline at a lower shortening velocity. The apparently anomalous result may be attributed to the difference in the protocols parameters used as the number of contractions (Tomas et al., 2010) and varied duty cycle and cycle time between the studies. The weight of evidence supports the notion that increasing velocities leads to increased fatigue, although a definitive assertion has yet to be made regarding the exact manner in which this occurs. For instance, a greater change has been observed in the contractile properties of muscle after concentric contraction than after isometric contractions, indicating a tendency for concentric tasks to be dominated by peripheral fatigue (Babault et al., 2006; Lanza et al., 2004). Isometric tasks have been found to be predominantly influenced by central mechanisms (Babault et al., 2006), yet others noted no decrease in voluntary activation (Callahan et al., 2009; Lanza et al., 2004). Again, the difference in duty cycle and cycle time between the protocols may have been responsible for the different observations. Babault et al. (2002) observed a decrease in neural drive for slower contractions of the knee extensors, likely attributed to an inhibition of the Ib afferents.

The high sensitivity of fatigue mechanisms to differences in protocol poses a challenge to deriving a precise relationship between the relative contributions of the causative mechanisms at any given velocity without standardized experiments. The majority of the studies do indicate, however, that one or more central mechanisms are likely dominant at lower shortening velocities and isometric actions, and that as the muscle shortening velocity increases, so does the contribution of peripheral mechanisms.

### 1.2 Fatigue mechanisms influence recovery

Rates of recovery from fatigue also vary depending on etiology. A rapid restitution of voluntary force after brief, high-intensity exercise has been observed and may be attributable to a recovery of central mechanisms within 2 min and certain peripheral aspects such as excitation-contraction coupling and muscle reperfusion within 5 min. Complete recovery, however, was found to take hours due to the prolonged peripheral impairment in intracellular Ca^2+^ release or sensitivity (Carroll et al., 2017).

### 1.3 Modeling fatigue

Mathematical models of LMF have sought to predict the decline in peak force/torque under various task conditions using many approaches. While a review of all the modeling techniques is beyond the scope of this paper, it may be mentioned that the most occupationally relevant models are computationally simple, require as input only data that is readily available, and can realistically depict the processes of fatigue and recovery under specified task conditions (Rashedi and Nussbaum, 2015).

#### 1.3.1 Compartment theory

Compartment theory, often used to model transport phenomena (Chladná et al., 2021; Görtz and Krug, 2022), has proved to be a particularly useful technique for modeling LMF using only the time varying target load (TL) as input. In an occupational setting, the simplicity of this method has the unique advantage of being able to generate predictions without using any biological measurements such as surface electromyography (sEMG), oxygen consumption, or carbon dioxide production. It was first used to model LMF by dividing muscles into three states: resting, active, and fatigued (Liu et al., 2002). In their approach, resting/recovered MUs could be recruited into the active state, which could then move into the fatigued state. Fatigued MUs were allowed to be directly activated when needed, but never allowed to return to the resting state. Complete recovery was precluded by this approach, but was addressed in the three compartment controller (3CC) model (Xia and Frey-Law, 2008).

#### 1.3.2 3CC model

The 3CC model rearranged the flow between the same 3 compartments so all MUs could return to the recovered state once the activation drive was switched off. Critically, it was also proposed that dynamic loads could be modeled by expressing the instantaneous desired torque as a fraction of the peak achievable torque at a given combination of joint angle and joint velocity. This would allow the model input (TL) to remain a fractional value, but would be calculated by looking up a 3-dimensional peak torque-velocity-angle (TVA) surfaces (Frey-Law et al., 2012a) using torque-velocity-angle data triplets from the activity. To our knowledge, this capability has not so far been validated.

In an update to the 3CC model, recovery during rest periods was enhanced in the 3CCr model to improve prediction accuracy for intermittent isometric tasks (Looft et al., 2018). A recently reported four-compartment muscle fatigue model (MFM) also distinguishes between the short-term and long-term fatigued states in isometric tasks (Michaud et al., 2024), but it stops short of extending to dynamic exertions.

The 3CCr model based on compartment theory has been validated against an extensive dataset of various isometric tasks (Frey-Law et al., 2012b; Looft et al., 2018; Looft and Frey-Law, 2020; Rakshit et al., 2022; Rakshit et al., 2021) and provides reasonable predictions for strength decline in short term isometric tasks (Michaud et al., 2024), both sustained and intermittent. However, it cannot distinguish between isometric and dynamic tasks and predicts identical torque declines for both as long as TL profiles are equal in both. This assumption may not always hold true. It also does not distinguish between the responsible fatigue/recovery mechanisms, which has the advantage of avoiding complexity so long as it only deals with one activity type. However, if it is to be applied to tasks with varying contraction velocities (such as through the use of TVA surfaces), the etiology of fatigue under each condition must be carefully considered and accounted for.

#### 1.3.3 Extending the 3CC model

With very few exceptions (Dalton et al., 2012), most studies have observed higher velocities to be correlated to lower fatigue (Bogdanis et al., 2007; Mathiassen, 1989; Matsuura et al., 2011; Morel et al., 2015; Perry-Rana et al., 2002; Tomas et al., 2010) albeit under varied task conditions (duty cycle (DC) and cycle time (CT)). Accordingly, it was hypothesized that joint velocity would affect joint strength while experimentally validating the 3CCr MFM.

In this paper, we will first investigate the relationship between joint velocity and fatigue through experimental data and compare it to 3CCr prediction results. Then a four-compartment controller model of muscle fatigue with enhanced recovery (4CCr) considering central and peripheral fatigue separately based on the original 3CCr will be developed for both static and dynamic tasks (Rakshit, 2023).

## 2 Materials and methods

### 2.1 Experimental data collection

#### 2.1.1 Participants

A total of 32 individuals (23 male, 9 female) participated this study (Table 1). Due to scheduling constraints, not all participants were able to contribute to the data for every joint. It was required that participants be healthy and have a BMI less than 30 but not less than 18.5. Participants were also screened to exclude professional athletes as training for a particular activity can skew the distribution of fiber types (Plotkin et al., 2021). Individuals with a history of neurological impairments or surgery in the tissue surrounding the respective joints were also excluded to prevent adverse outcomes in these potentially vulnerable populations. All participants were prohibited from participating in intense activity for the entire duration that they were enrolled in the study. The study was approved by the Institutional Review Board of Texas Tech University and participants signed informed consent forms that provided details of the study.

**TABLE 1:** Participant data by joint for the experiment.

Joint	Number of participants of each sex	Height (cm) Mean (SD)	Weight (kg) Mean (SD)	Age (years) Mean (SD)
Elbow	13M, 7F	170 (10)	70 (12)	24 (3)
Shoulder	14M, 4F	170 (10)	70 (10)	25 (5)
Hip	13M, 5F	171 (10)	70 (13)	23 (3)
Knee	14M, 4F	170 (10)	70 (10)	25 (5)

Height, weight, and age are noted as mean (standard deviation).

#### 2.1.2 Protocol

**FIGURE 1:**
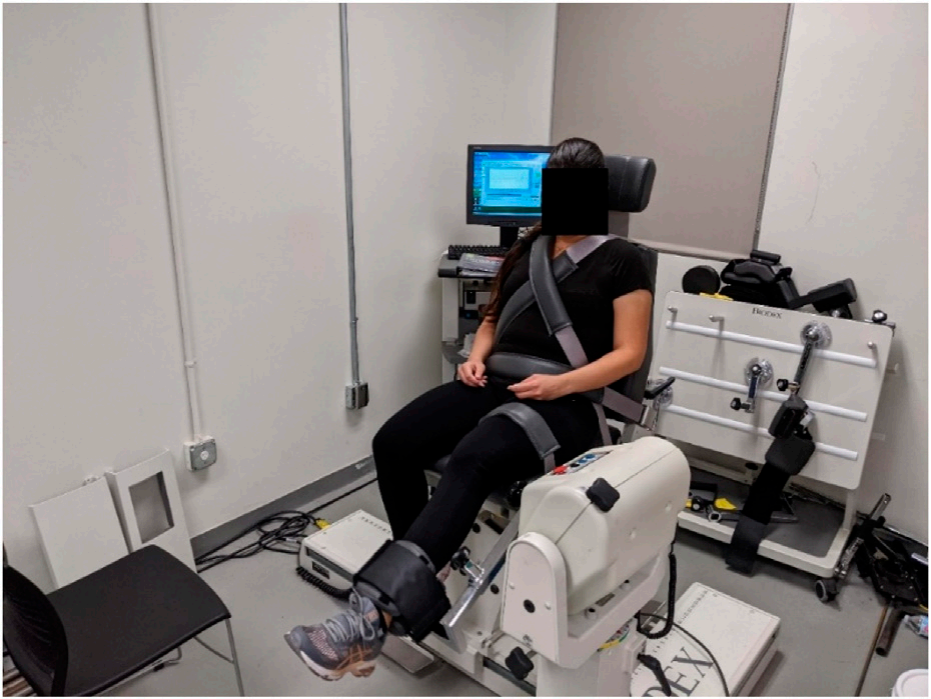
Experimental setup.

Torque, velocity, and angle data were collected using a Biodex System 4 Pro Dynamometer (Biodex Medical Systems Inc., Shirley, NY, United States) (Figure 1) at 100 Hz and stored within a custom MATLAB (The MathWorks Inc., Natick, MA, United States) data structure for further processing. Each participant completed the experiment in 1 training session and 5 test sessions. A minimum of 48 h separated consecutive test sessions, but training and test sessions were allowed to be on consecutive days. During the training session they were familiarized with the equipment and protocol and trained to perform timed maximal voluntary isometric contractions (MVIC) at the experimenter’s instruction. In the test sessions that followed, they performed the test protocol at a predetermined isokinetic velocity. The order of velocities used was randomized between participants to minimize order effects.

**FIGURE 2:**
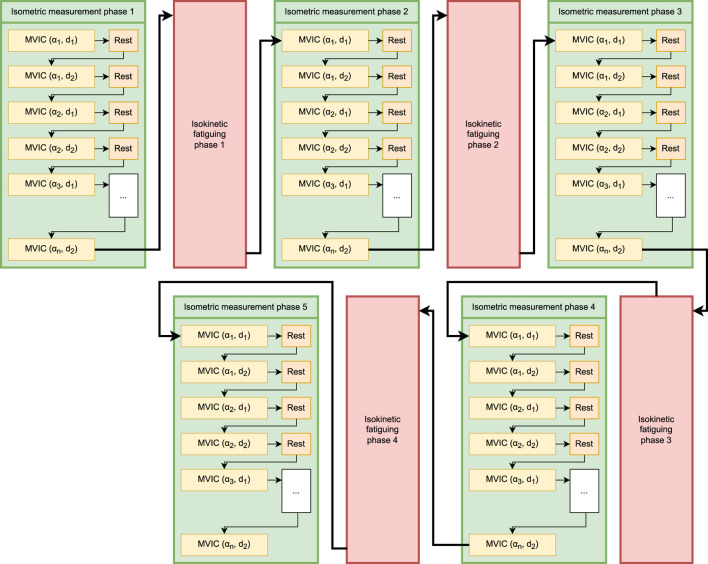
Schematic of the fatiguing protocol. Green boxes represent isometric measurement phases, and pink boxes represent isokinetic fatiguing phases. *n* MVICs are performed within each isometric phase: $\alpha_{i}$ denotes the i -th ROM stop, and $d_{j}$ denotes the j -th rotation direction where $j\in \left\{1,2\right\}$ .

**FIGURE 3:**
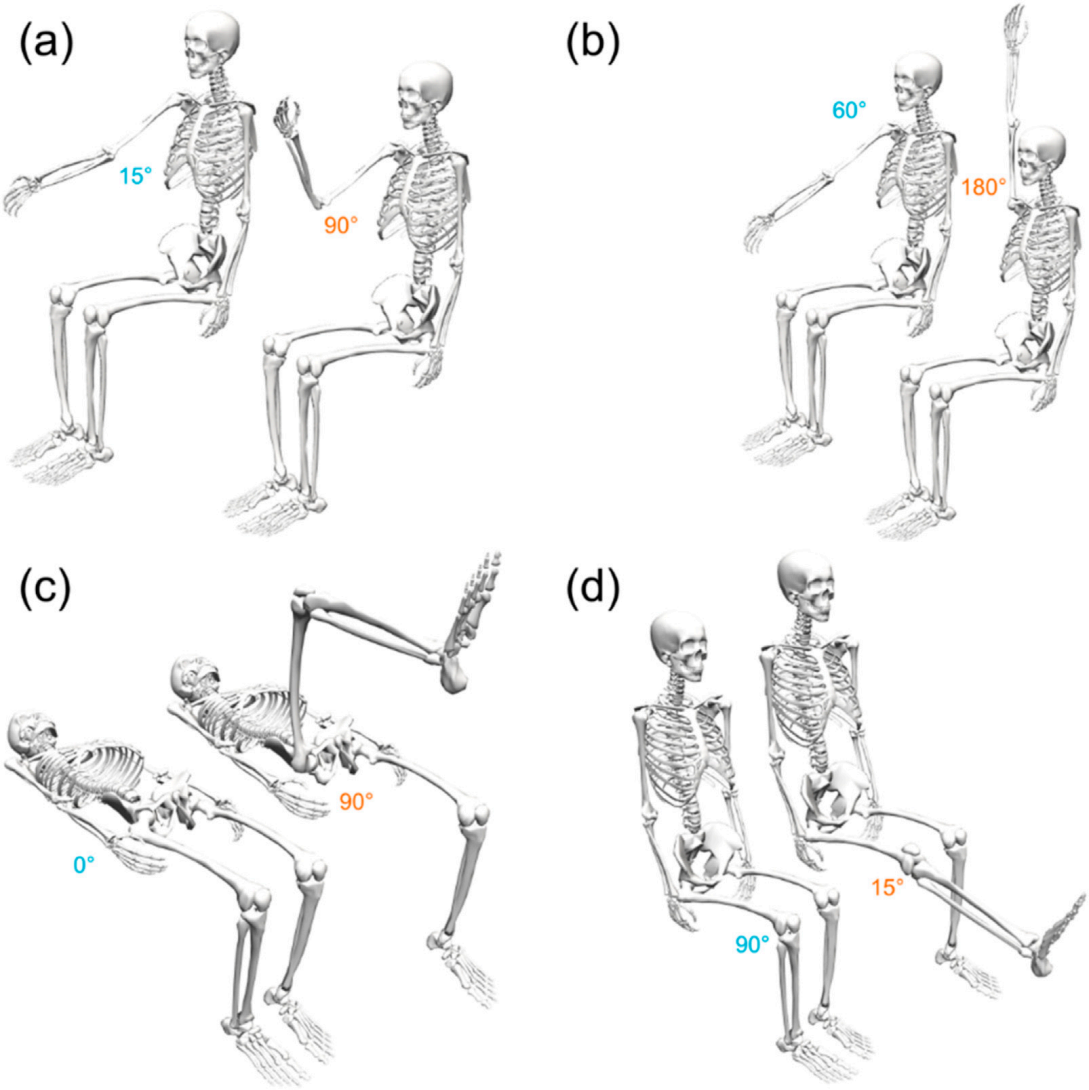
The initial (blue) and final (orange) ROM stops for the **(A)** elbow, **(B)** shoulder, **(C)** hip, and **(D)** knee joints. All poses depicted here using the right limbs of a generic skeletal model; the corresponding ROM stops for the left limbs are symmetric about the sagittal plane.

A test session consisted of alternating bouts of isometric and isokinetic activity over the same predetermined ROM (Figure 2). During the isometric phase, the participant’s dominant limb was positioned sequentially at multiple joint angles (ROM stops), and they were instructed to perform a 3-s MVIC in the flexion (or extension) direction, followed by 2 s of rest and then a 3-s MVIC in the opposite direction, after which the dynamometer arm moved their limb to the next ROM stop. After the final MVIC at the last stop, the dynamometer switched to isokinetic mode for 60 s, restricting the maximum joint velocity to a predetermined value. The participant was instructed to exert maximum effort in this phase as well while flexing and extending the limb between the limits of the ROM (Figure 3). The dynamometer would then again switch to the isometric mode to measure MVICs. Electromyography was omitted as it would have been impossible to ensure the same motor units were consistently in the detection volume of the sensors while the participants’ limbs moved through the range of motion (De Luca, 1997). Five isometric measurement phases (ISOM1 through ISOM5) and four isokinetic fatiguing phases (ISOK1 through ISOK4) were conducted for every participant.

ROM limits and velocities used for all subjects during the experiment (Table 2) were determined from a pilot study with 4 participants. The values chosen from the pilot study were expected to be achieved by all members of the target population, and all participants of the main study were ultimately able to achieve the selected ROM limits and velocities. The number of ROM stops for each joint were chosen to minimize the time spent in the isometric phase while still obtaining readings over the entire ROM.

**TABLE 2:** Isometric ROM stops and velocities tested in the main experimental protocol. The minimum and maximum values of the ROM stop demarcate the mechanical ROM limits tested for each joint.

Joint	ROM (°)	Isometric ROM stops (°)	Isokinetic velocities (°/s)
Elbow	75	15, 30, 45, 60, 75, 90	20, 30, 45, 60, 90
Shoulder	120	60, 90, 120, 150, 180	20, 30, 45, 60, 75
Hip	90	0, 15, 30, 60, 75, 90	30, 45, 60, 90, 120
Knee	75	90, 75, 60, 45, 30, 15	30, 60, 90, 120, 150

### 2.2 Data processing

Peak isometric torque values over time, measured before and after every minute of isokinetic activity, were used to estimate the extent of fatigue. For each functional muscle group (FMG) tested by a single participant in each session, the raw peak torque values in each isometric phase were normalized by the maximum value across all joint angles in that iteration, yielding 5 sets (corresponding to 5 isometric measurement phases) of n values each (corresponding to MVICs measured at n ROM stops) between 0 and 1. As each set was normalized by its own maximum, every set represented the variation in normalized strength over joint angle. The 5 curves thus obtained for a single subject during one session were averaged to obtain one subject-specific normalized torque-angle plot for that session. To obtain a single representative value of strength for each iteration, the normalized torque-angle curve was subsequently scaled to fit a simple majority of points of each raw isometric measurement using a linear least-squares method. For a joint in which MVICs were measured at n ROM stops, all combinations of $\frac{\left\lceil n+1\right\rceil }{2}$ data points were used to evaluate a scaled fit, and the scale factor corresponding to the combination producing the lowest summed residuals was chosen to represent that iteration. The difference between the representative scale factors for consecutive isometric iterations was used as a measure of strength decline during the intervening 60-s isokinetic task. The amount of recovery during the isometric phases was assumed to be negligible, so any decreases in strength could be attributed to the isokinetic activity alone.

### 2.3 Statistical analysis

The Shapiro-Wilk test was used to assess the normality of the torque decline data. The results for 24 of the 200 distributions (8 functional muscle groups × 5 velocities × 5 iterations) were significant, indicating non-normality for those 24 distributions. The remaining data, comprising 88% of the dataset, were found to be normally distributed.

Flexor and extensor peak torques were measured separately for each joint, so each participant generated two sets of fatigue data for each joint that they exercised. The fatigue rate for each FMG × velocity × iteration combination was averaged across all participants and the standard deviation was calculated in each case. A two-way ANOVA with a type I error level of 0.05 was run on the resulting dataset to determine whether any differences observed were statistically significant. Within each FMG × iteration combination, the effect of velocity was determined by performing a simple linear regression.

### 2.4 Simulation

The experimental fatiguing protocol described above was modeled in SIMULINK (The Mathworks, Inc., Natick, Massachusetts. United States) as alternating sets of activities, with task parameters (TL, DC, CT) for the isometric phase coded as (1, 0.2, 15 s), and for the isokinetic phase coded as (1, 0.5, $\frac{2\times ROM}{V}$ ) with a total simulation time of 690 s. The 3CCr model was also implemented in SIMULINK with model parameters for the generalized elbow, shoulder, and knee joints drawn from (Frey-Law et al., 2012b; Looft et al., 2018). As hip-specific parameters are not yet available, generalized fatigue parameters were used for simulations.

#### 2.4.1 Four-compartment controller with enhanced recovery (4CCr) model of fatigue

**FIGURE 4:**
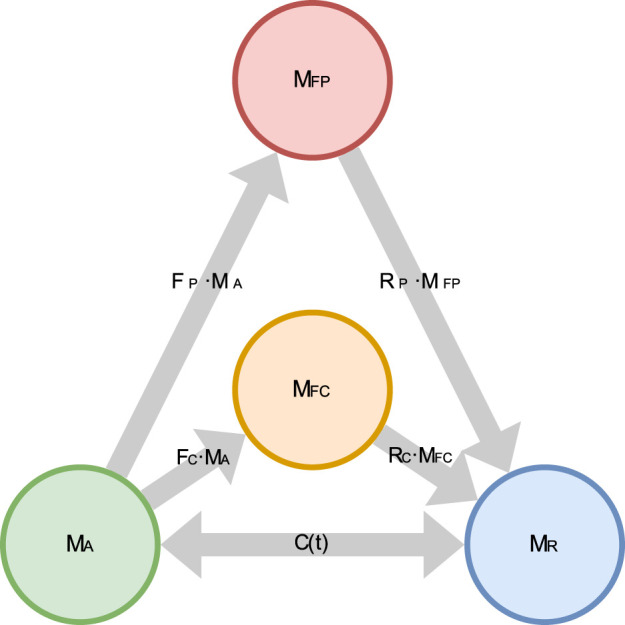
Schematic diagram of the 4CCr model depicting the flow of MUs between the 4 states.

In the 3CCr model, active MUs would be allowed to pass into a single fatigued state. In our 4CCr model, we divide the fatigued compartment of 3CCr into two: peripherally fatigued, and centrally fatigued. Accordingly, the governing equations for the four compartments depicted in Figure 4 are adapted from those describing the 3CCr model (Looft et al., 2018; Xia and Frey-Law, 2008) as Equations 1–4:

$$\frac{dM_{A}}{dt}=-F_{P}M_{A}-F_{C}M_{A}+C\left(t\right)$$ (1)

$$\frac{dM_{R}}{dt}=R_{P}M_{FP}+R_{C}M_{FC}-C\left(t\right)$$ (2)

$$\frac{dM_{FP}}{dt}={-R}_{P}M_{FP}+F_{P}M_{A}$$ (3)

$$\frac{dM_{FC}}{dt}={-R}_{C}M_{FC}+F_{C}M_{A}$$ (4)

where $M_{A}$ , $M_{R}$ , $M_{FC}$ , and $M_{FP}$ are the sizes of the active, resting, centrally fatigued, and peripherally fatigued compartments, respectively, expressed as the fraction of all MUs occupying that particular state at a given time. The size of each compartment represents the fraction of the total force capacity of the muscle that is either in use (active), available for immediate use (resting), or unavailable for use (centrally fatigued, peripherally fatigued).

$C\left(t\right)$ retains its definition from 3CCr as a bidirectional activation/deactivation drive that serves to transition MUs between the active and resting states depending on the instantaneous requirement of the task as in Equation 5:

$$C\left(t\right)=L\times \min\left(TL-M_{A},M_{R}\right)$$ (5)

where L is a force development/relaxation factor that ensures the developed force quickly and closely tracks TL . Extremely small values can cause poor tracking, but any value greater than ∼10 is sufficient to ensure that the model responds to changes in TL quickly. As the predictions have a very low sensitivity to L , it was arbitrarily set to 20 for all subsequent simulations.

Each of the new fatigued compartments has its unique set of associated F and R values corresponding to the expected relative rates of fatigue and recovery due to peripheral and central mechanisms, respectively. The recovery rate constant for peripheral fatigue $R_{P}$ is assumed to have a constant value $R_{P_{0}}$ throughout as in Equation 6:

$$R_{P}=R_{P_{0}}$$ (6)

In accordance with 3CCr, $R_{C}$ assumes one of two discrete values depending on whether TL is non-zero or not as described by Equation 7:

$$R_{C}=\left\{\begin{array}{l} R_{C_{0}}\;if\;TL=0\\ rR_{C_{0}}\;if\;TL>0 \end{array}\right.$$ (7)

where $R_{C_{0}}$ is the baseline recovery rate constant for central fatigue, and r is the recovery rate multiplier for rest. $F_{P}$ and $F_{C}$ vary continuously with velocity V . During an isometric task ( $\left.V=0\right)$ , $F_{C}=F_{C_{0}}$ and $F_{P}=0$ . With increasing velocity $F_{P}$ increases while $F_{C}$ decreases according to Equations 8, 9:

$$F_{P}=F_{P_{0}}\left(1-e^{-kV}\right)$$ (8)

$$F_{C}=F_{C_{0}}e^{-kV}$$ (9)

where k is the velocity coefficient having the units s/°.

#### 2.4.2 Parameter estimation

The introduction of a peripherally fatigued compartment into the 3CCr model requires the evaluation of three additional variables: $F_{P}$ , $R_{P}$ , and k . The first two variables control the baseline rate of flow of MUs into and out of the peripherally fatigued compartment, and k determines how the velocity of contractions modifies the base rate of flow of MUs. To determine the values of the new parameters that best described the experimental dataset collected and post processed in Section 2.2, the 4CCr model was recreated in Simulink with the three new parameters as base workspace variables. Values for these parameters were iteratively generated by a MATLAB genetic algorithm and were, in turn, used to generate model predictions. The solver was constrained to look for a solution within a maximum of 24 h on clusters at Texas Tech University High Performance Computer Center and to terminate if the average change in the fitness function over 300 generations was less than or equal to the default FunctionTolerance. In each case, the optimization completed well within 24 h when the function tolerance stopping criterion was met. The optimized parameter values are obtained based on 14 subjects and listed in Table 3.

**TABLE 3:** Optimized parameter values for the 4CCr model.

Muscle group	k	$R_{P}$	$\frac{F_{P}}{R_{P}}$
Shoulder flexors	−0.0086	1.2E-5	1,211
Shoulder extensors	−0.0233	8.2E-6	884
Hip flexors	−0.0254	1.0E-5	848
Hip extensors	−0.0319	3.2E-5	552
Knee flexors	−0.0182	1.0E-5	927
Knee extensors	−0.0147	2.2E-5	223

## 3 Validation and results

### 3.1 Results for fatigue rate depending on joint velocity

**FIGURE 5:**
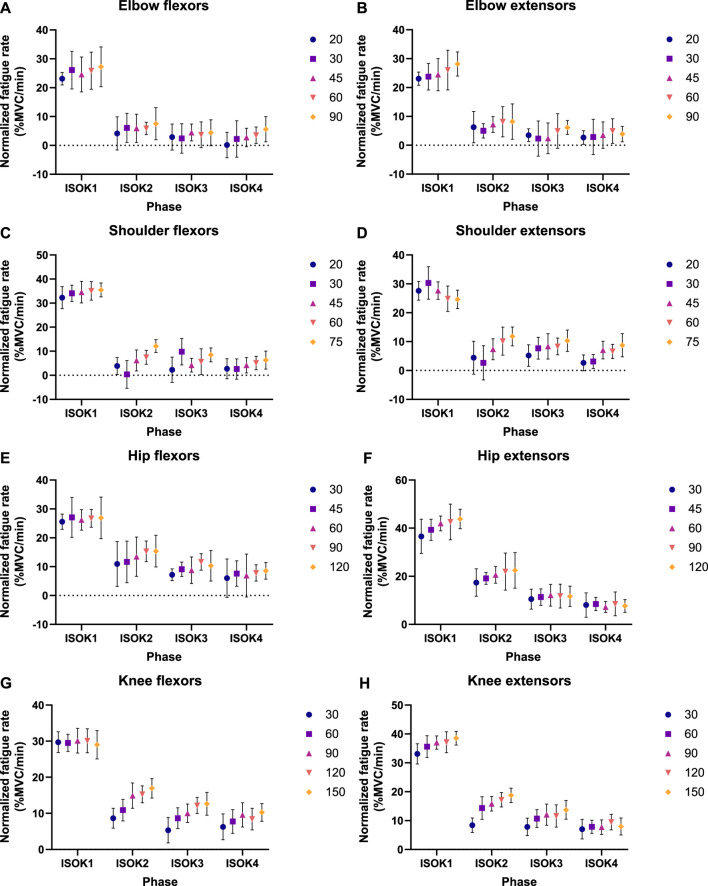
Fatigue rates as experimentally measured by decline in normalized peak isometric strength per minute of isokinetic activity for **(A)** elbow flexors, **(B)** elbow extensors, **(C)** shoulder flexors, **(D)** shoulder extensors, **(E)** hip flexors, **(F)** hip extensors, **(G)** knee flexors, and **(H)** knee extensors. Data points represent mean normalized fatigue rates, and error bars indicate the standard deviation. A different set of velocities was used during the isokinetic phase for each joint, given by the legend in °/s.

**FIGURE 6:**
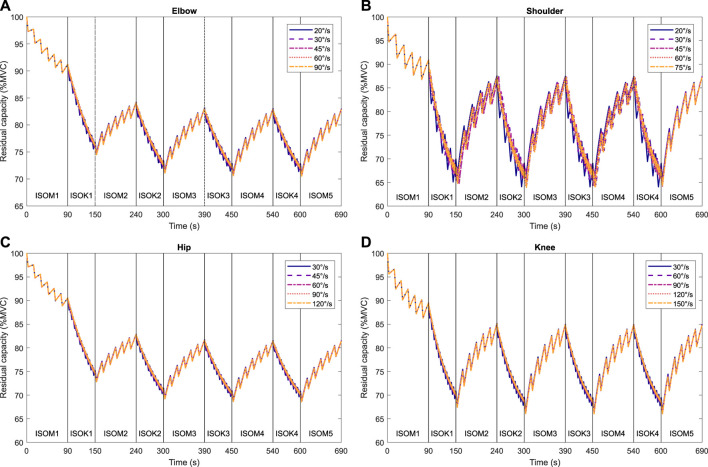
Predictions of residual capacity during alternating intermittent isometric and intermittent isokinetic tasks at different velocities according to the 3CCr model: **(A)** elbow, **(B)** shoulder, **(C)** hip, and **(D)** knee joints. Joint-specific model parameters are used for the elbow, shoulder, and knee joints, and generalized parameters are used for the hip joint (Frey-Law et al., 2012b; Looft et al., 2018). All parameters are for a general population.

Isometric strength is observed to decline after every isokinetic phase (Figure 5) in the experimental data. These experimental results are qualitatively in accordance with the 3CCr model predictions till ISOM2 (Figure 6) which also predicts a lower strength for ISOM2 as compared to ISOM1. The experimental and predicted trends diverge thereafter, with ISOM3 through ISOM 5 showing consecutive losses within the experimental data, while the predicted strengths at the end of each of those phases remain nearly unchanged due to extensive recovery during large rest periods afforded within each isometric phase. The loss in strength during consecutive isokinetic phases grows smaller as the activity progresses but is predicted to be negligible by the 3CCr model after ISOM2.

**FIGURE 7:**
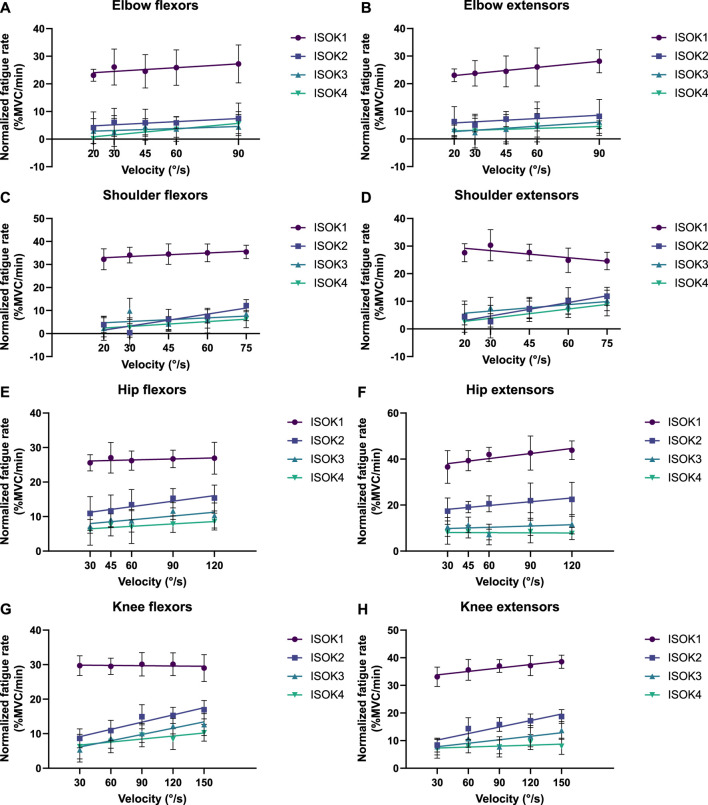
Decline in peak isometric strength per minute of isokinetic activity performed as a function of isokinetic task velocity for **(A)** elbow flexors, **(B)** elbow extensors, **(C)** shoulder flexors, **(D)** shoulder extensors, **(E)** hip flexors, **(F)** hip extensors, **(G)** knee flexors, and **(H)** knee extensors. Data points represent mean normalized fatigue rates, and error bars indicate the standard deviation. Each solid line depicts the strength decline in a different phase of the fatiguing protocol (ISOK1-ISOK4) by the change in velocity.

Isometric strength is also affected by the velocity of isokinetic phase, with higher velocities resulting in correspondingly greater losses of strength (Figure 7; Table 4) for all muscle groups except the elbow flexors. In contrast, the 3CCr model predicts no difference in isometric strength due to differences in isokinetic testing velocity alone for any joint, with the strength curves overlapping almost entirely (Figure 6).

**TABLE 4:** Linear regression results for fatigue rate in each isokinetic phase. Values for slope are the change in normalized fatigue rate (%MVC/min) divided by the change in angular velocity (°/s), with the values in parentheses indicating the 95% confidence interval bounds.

Functional muscle group	ISOK1		ISOK2		ISOK3		ISOK4	
	Slope (%MVC s/° min)	p-value	Slope (%MVC s/° min)	p-value	Slope (%MVC s/° min)	p-value	Slope (%MVC s/° min)	p-value
Elbow flexors	0.04514 (−0.002074 0.09235)	0.061	0.03793 (−0.000854 0.07671)	0.055	0.02422 (−0.01068 0.05911)	0.172	0.07004 (0.03494 0.1051)	<0.001*
Elbow extensors	0.07383 (0.03448 0.1132)	<0.001*	0.03937 (0.001944 0.07679)	0.039*	0.04905 (0.01115 0.08695)	0.012*	0.02241 (−0.01148 0.05631)	0.192
Shoulder flexors	0.05142 (0.01064 0.09219)	0.014*	0.1744 (0.1286 0.2201)	<0.001*	0.5265 (−0.001729 0.1070)	0.058	0.07012 (0.03213 0.1081)	<0.001*
Shoulder extensors	−0.08559 (−0.1293–0.04184)	<0.001*	0.07661 (0.1113 0.2136)	<0.001*	0.07661 (0.03731 0.1159)	<0.001*	0.1117 (0.07940 0.1441)	<0.001*
Hip flexors	0.009609 (−0.01277 0.03199)	0.396	0.05328 (0.02652 0.08005)	<0.001*	0.03682 (0.01801 0.05562)	<0.001*	0.02328 (0.0004920 0.04606)	0.045*
Hip extensors	0.07307 (0.03735 0.1088)	<0.001*	0.05474 (0.01800 0.09147)	0.004*	0.01847 (−0.01069 0.04762)	0.212	−0.002305 (−0.02638 0.02177)	0.850
Knee flexors	−0.002833 (−0.01860 0.01293)	0.722	0.06983 (0.05531 0.08436)	<0.001*	0.06063 (0.04613 0.07513)	<0.001*	0.02900 (0.01328 0.04472)	<0.001*
Knee extensors	0.04143 (0.02573 0.05714)	<0.001*	0.07847 (0.06327 0.09367)	<0.001*	0.04217 (0.02425 0.06008)	<0.001*	0.01153 (−0.002398 0.02546)	0.104

Significantly non-zero slopes are denoted by a * next to the corresponding p-value.

### 3.2 4CCr model validation

When the velocity is 0, the 4CCr equations collapse into those describing 3CCr and the resulting outputs are identical. 4CCr, therefore, generates the same predictions for isometric tasks (whether sustained or intermittent) as its predecessor.

**FIGURE 8:**
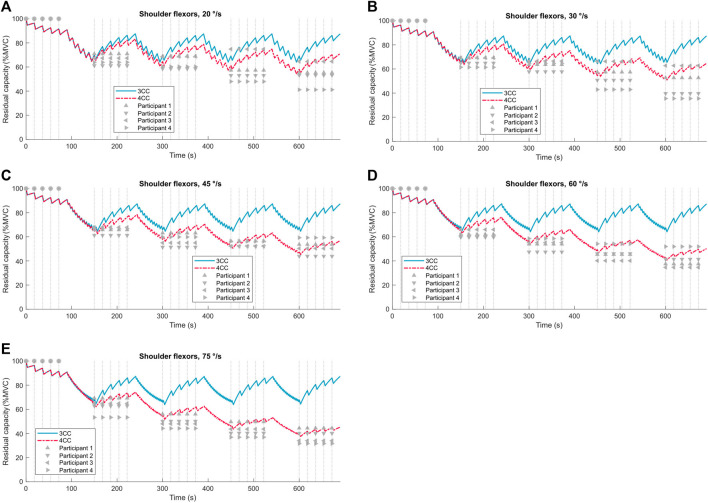
3CCr (blue) and 4CCr (red) predictions of fatigue for the shoulder flexors compared to experimental data from four participants (grey triangles) at angular velocities ranging from 20 °/s to 75 °/s **(A–E)**. Vertical dotted lines mark sample times.

When the velocity is not zero, the model predictions are compared against the experimental data from the 4 participants whose data was not used to develop the model parameters in Table 3. Data from 4 subjects were set aside for validation, and the remaining were used for model fitting with no crossover. Torque decline values are compared against both the 4CCr and 3CCr model predictions for all joints except the elbow in Figure 8 and Supplementary Figures S1–S5 in the Supplemental Material. The availability of torque data at multiple known sample times throughout the duration of the fatiguing task enables the calculation of Pearson’s correlation coefficients, reported in Tabs. 5 and Supplementary Tables S1–S5 in the Supplemental Material.

**TABLE 5:** Pearson’s correlation coefficients of 3CCr and 4CCr predictions for shoulder flexors to experimental data from four participants.

Joint velocity (°/s)	20		30		45		60		75	
Participant	3CC	4CC	3CC	4CC	3CC	4CC	3CC	4CC	3CC	4CC
1	0.69	0.91	0.68	0.94	0.71	0.96	0.69	0.97	0.68	0.98
2	0.73	0.90	0.65	0.94	0.70	0.95	0.71	0.95	0.69	0.97
3	0.71	0.83	0.72	0.91	0.69	0.96	0.65	0.98	0.67	0.98
4	0.68	0.92	0.64	0.93	0.72	0.93	0.72	0.93	0.71	0.97

In the data processing pipeline, torque values throughout the ROM were used to determine strength during an isometric measurement phase, and, as a result, a single value of strength is obtained for each ISOM phase. For comparison with model predictions at each time an isometric measurement was performed, the same constant value of strength is used for 6 (hip, knee) or 5 (shoulder) times within an isometric phase.

Pearson’s correlation coefficients are calculated for both sets of predictions against data from each of the four participants: Table 5 is for the shoulder flexors and Supplementary Tables S1–S5 in the Supplemental Material are for other joints.

## 4 Discussion

The hypothesis that velocity of contraction would affect isometric strength measurements was successfully confirmed for 7 of the 8 muscle groups studied, with only negligible velocity effects noted for the elbow flexors (Figure 7). Fatigue was noted to increase nearly linearly with increasing velocity (Table 4), indicating that the greater power expenditure associated with higher velocities may have been responsible for increasing strength losses. A relatively narrow range of velocities chosen for the experiment may explain why no effects were observed for the elbow flexors, and it may well be the case that the increase in fatigue rates is non-linear if higher velocities are also considered.

The trend of increasing fatigue rate with higher velocities has a few possible explanations. Velocity within a fixed ROM is inversely proportional to the CT, resulting in successively shorter CTs for higher velocities. Shorter CTs allow decreased time for muscle excitation and relaxation, and this has been proposed to be a responsible mechanism for reduced strength at higher velocities (Tomas et al., 2010). Peak torque may also have decreased with increasing velocities due to a shift in the relative contributions to torque production from both Type I and Type II fibers at low velocities to predominantly Type II fibers at higher velocities (Perry-Rana et al., 2002).

EMG was not measured for this study, but since the model itself deals with contributions of central and peripheral fatigue, a discussion of potential observations is warranted. Peripheral fatigue typically manifests in decreased conduction velocities and as larger, fast-twitch motor units dropping out, resulting in a shift to lower frequencies in the power spectrum. The markers for central fatigue are distinct: As recruitment and firing rates increase to compensate for declining force output, an increase in the amplitude of the signal may be observed. Provided a suitable method is agreed upon for measuring EMG from the same set of motor units throughout isokinetic and isometric tasks, these key observations may be used to further calibrate the model parameters or validate its predictions.

The 3CCr model, owing to its ease of use, may still be employed unaltered in conjunction with appropriate TVA surfaces to model fatigue for dynamic tasks (including isokinetic tasks), but will not predict velocity effects. If the distinction between possible contraction velocities that an activity may be performed at is important, the model will likely require modification to account for those differences.

The 4CC model of LMF described here uses joint angular velocity along with target load to determine the relative contributions of central and peripheral mechanisms. Low velocities are dominated by central mechanisms, and higher velocities allow peripheral mechanisms to assume a greater role. In its present formulation, all muscle properties are noted for an agonist muscle group in concentric actions, and the velocities referred to are the joint angular velocities resulting in concentric action (muscle shortening) of the agonists.

In maximal voluntary concentric contractions, the shortening agonistic group would be responsible for the bulk of the motive force (Hirono et al., 2022). TL for the antagonistic group is therefore considered 0 for simplicity, and that for the agonistic group is non-zero. In reality, antagonistic muscles provide stability and fine motor control in concentric actions of the agonists, so their contribution to the total torque output may not be exactly nil. As the model allows low intensity activities to continue almost indefinitely, the low TL stabilizing activity of the antagonists would not serve as a bottleneck and predict earlier fatigue for the activity as a whole. RC predictions would thus not be significantly altered. However, for fine-grained modeling of the contributions of both agonistic and antagonistic groups for a certain activity, experimental data on antagonistic force production in response to synergist contraction or co-contraction indices (Li et al., 2021) may be used to simultaneously drive two simulations—one for each muscle group. Such an approach would be particularly well-suited for incorporation in a joint-based musculoskeletal model.

Being an extension of the 3CC model, the new model reproduces its predictions exactly given the same task parameters and boundary conditions for isometric actions. This is important to ensure that in attempting to model more complex tasks with non-zero velocities, it does not lose its ability to accurately predict fatigue in zero-velocity tasks. It also ensures that the new model does not need to be revalidated for the trivial case as the 3CC model has already been validated against extensive experimental data.

The 3CC model was originally presumed to be exclusively representative of peripheral fatigue (Xia and Frey-Law, 2008), but later studies (Carroll et al., 2017; Morel et al., 2015) have indicated that central processes may contribute much more to isometric tasks than peripheral ones alone. Additionally, the extremely rapid recovery observed in the predictions of 3CC, especially during rest, is more characteristic of central mechanisms than peripheral ones, suggesting that it may in fact be predicting central fatigue at least for intermittent isometric tasks. This does not detract from its general accuracy for the conditions it was validated against, and in borrowing from its general structure, 4CC retains the same accuracy while renaming the only fatigued compartment in the previous model as the centrally fatigued compartment in the new model. The 4CC model’s key contribution lies in the provision of a second fatigued state that allows a more nuanced consideration of the underlying fatigue/recovery phenomena while still requiring no physiological measurements. As more organized data from fundamental research into the dependence of fatigue mechanisms on tasks parameters is available, Equations 6–9 can easily be updated within the framework of this model to reflect the latest understanding of the physiology of fatigue.

Correlation coefficients for the 4CCr model are consistently higher than those for the 3CCr model across all velocities and participants. Except for 3 cases with R-values between 0.9 and 0.8 (out of 120), R-values for 4CCr are >0.90 in all cases. Referring to Figure 8 and Supplementary Figures S1–S5 in the Supplemental Material, it is clear that these high correlation coefficients do not necessarily imply accurate predictions for the fatigued strength for every individual. Correlation coefficients are consistently >0.97 for both hip flexors and extensors, and this can be attributed to the negligible recovery predicted during the isometric phase being a much closer reflection of the same assumption during data processing. Indeed, since general parameters derived from a homogeneous sample are used, predictions are representative of the sample and not necessarily of any particular individual. However, a strong linear correlation is implied between 4CC predictions and experimental data, likely a result of the model’s ability to restrict recovery during periods of rest immediately following intense dynamic activity. While subject-specific parameters hold the promise of increased personalized prediction accuracy, deriving those parameters may prove to be a significant challenge using the processes outlined in this work. Nevertheless, these generalized parameters for the new model depict an improved ability to chart the progress of fatigue for alternating dynamic and isometric tasks.

It must be noted that validation for the 4CCr model need not be limited to isokinetic tasks. Activities in which the joint velocity follows a known pattern, such as isotonic contractions performed on a dynamometer, or bike ergometry (Bini et al., 2023; Trumbower and Faghri, 2005), can also be used to drive parameter estimation in conjunction with load cells or custom force-measurement apparatus. Once parameters for isokinetic activities have been reasonably well established, they may be further tuned to accommodate dynamic activities in which velocity changes continuously.

Though the 4CCr model provides a flexible framework to predict fatigue for a variety of commonplace tasks, it is not without its limitations. It approximates the effects of velocity on muscular function as currently understood but does not account for joint angle. Whereas extrafusal fibers in mammalian muscles are responsible for force production, intrafusal fibers provide the motor system continuous feedback about the length of the muscle through nuclear chain and static nuclear bag fibers, and about the rate of change of muscle length through dynamic nuclear bag fibers. Only the latter feedback is currently incorporated as the rate of muscle shortening can be directly correlated to joint angular velocity. Muscle length itself is indicative of joint angle, and the model is not capable of reproducing any angle-specific data, such as the influence of different ROM limits on fatigue rates if all other task parameters are kept the same. Further investigation may determine if integration of joint angle data into the model significantly improves predictions beyond simply referring to the appropriate TVA surface.

Some important limitations in the methods employed in this work must be noted. First, a small sample size of 14 was used to estimate all fatigue rates and velocity effects, while a larger one could have strengthened the findings. Moreover, female participants formed only 22%–35% of the study population for each joint, leading to an underrepresentation of women in the data. Given sufficiently large sample sizes, the influence of sex on fatiguability may be inferred from collected data (Avin et al., 2010; Rakshit et al., 2022; Rakshit et al., 2021), but given the limited number of participants in the cohort, the decision was made to combine data from male and female participants to maintain statistical power. Although the safe physical joint angle limits of the human body are typically greater than the ROM limits used here, smaller limits for high intensity and high velocity exercise can protect joints from overextension injury. Real world tasks are less likely to have externally imposed constraints on ROM, but to compensate the performer of the task may tend to self-regulate ROM to avoid injury. Therefore, while the full ROM has not been studied here, the results should still be applicable to most situations requiring near maximal effort at high velocity. Additional work may be required to model the progress of fatigue during isokinetic tasks at submaximal efforts and/or higher velocities. Furthermore, although the new 4CCr model was derived based on the data collected from a limited number of subjects, it could be further extended to certain specific populations for use in specialized applications.

## Data Availability

The raw data supporting the conclusions of this article will be made available by the authors, without undue reservation.
