# Supplementary material for: A four-compartment controller model of muscle fatigue for static and dynamic tasks
Source: Front Physiol. 2025 Feb 12;16:1518847. doi: 10.3389/fphys.2025.1518847 (PMC11861306; doi:10.3389/fphys.2025.1518847)
Supplement: Supplementary file 1 [file Table1.DOCX]

**Supplemental Materials**

Torque decline values are compared against both the 4CCr and 3CCr model predictions for all joints except the shoulder flexors, in Figure S1-S5. The availability of torque data at multiple known sample times throughout the duration of the fatiguing task enables the calculation of Pearson’s correlation coefficients except the shoulder flexors, reported in Tables S1-S5.


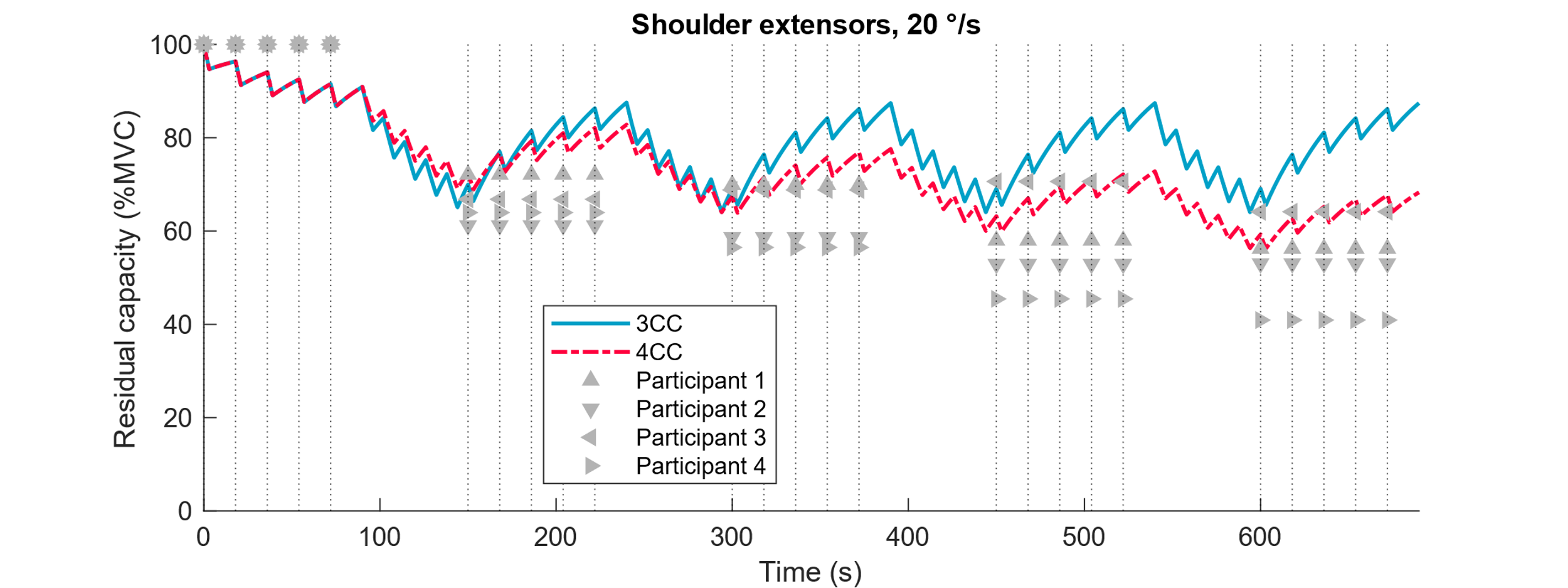


(a)


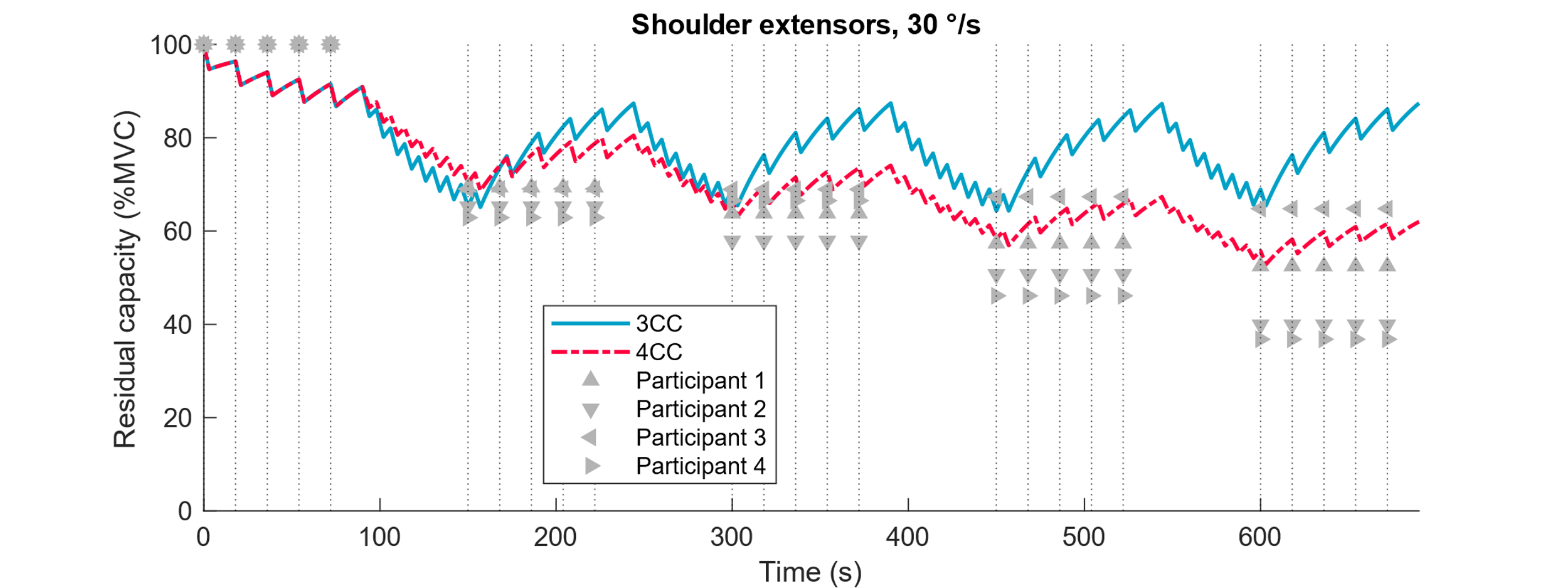


(b)


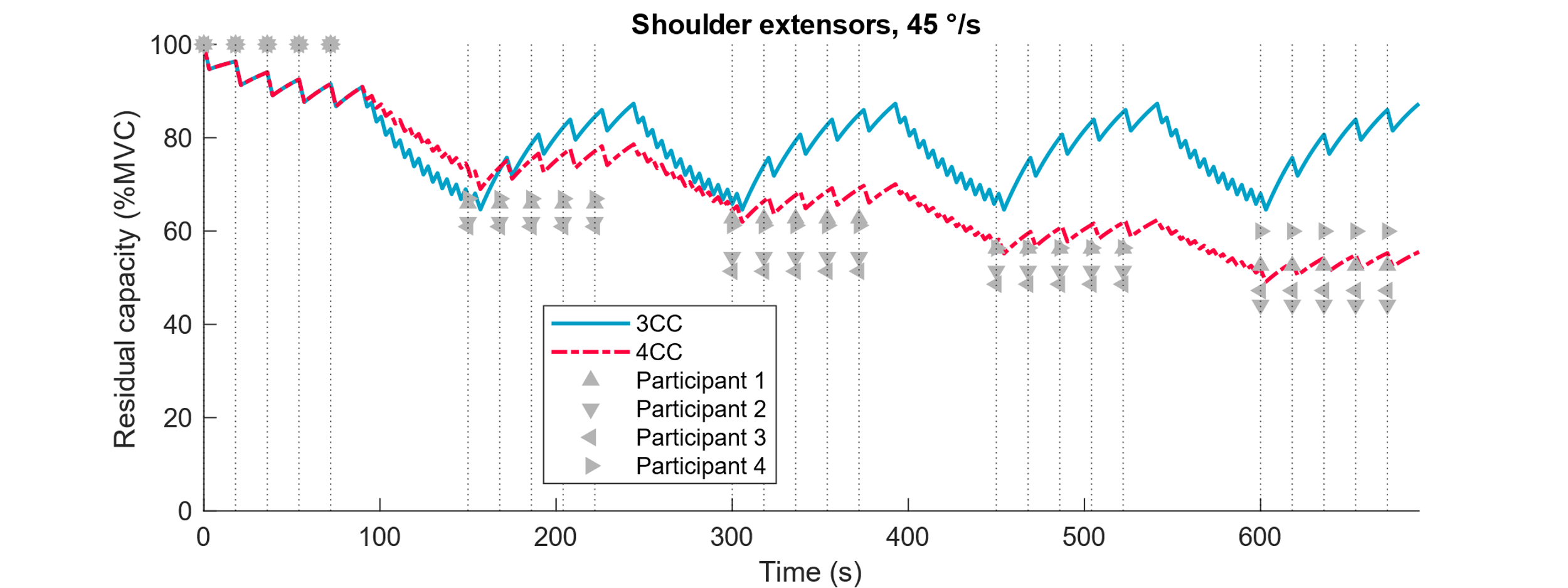


(c)


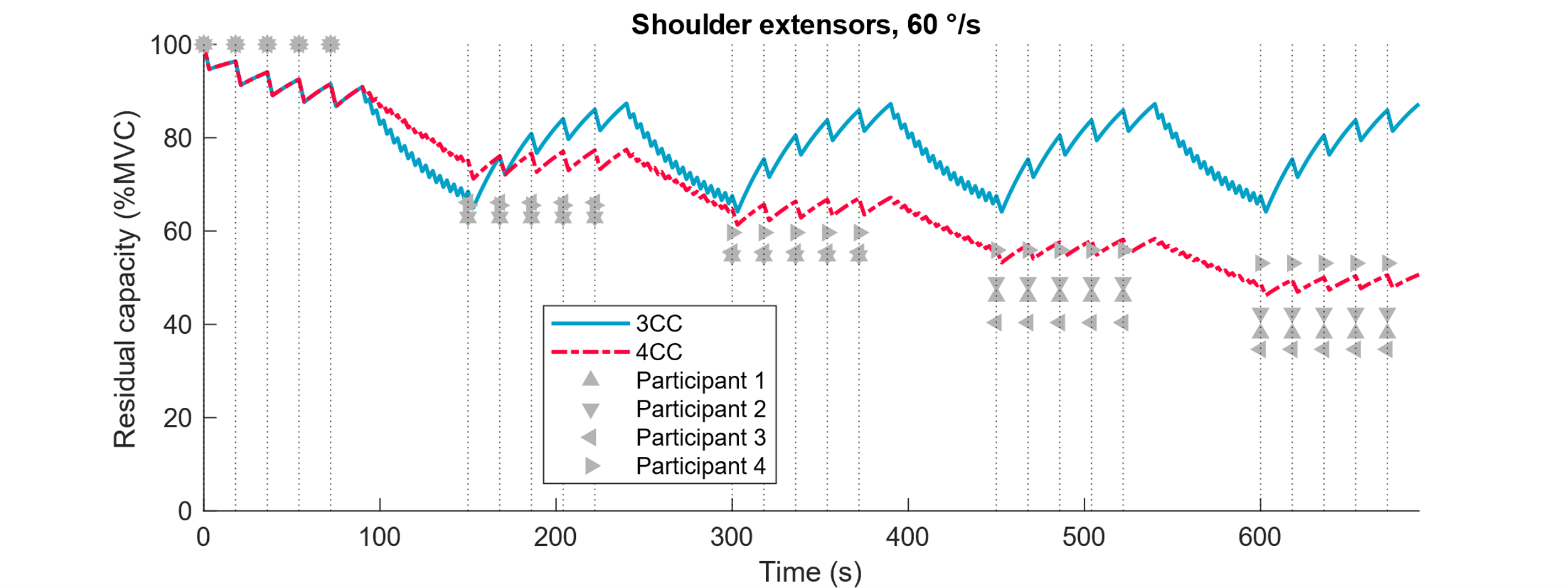


(d)


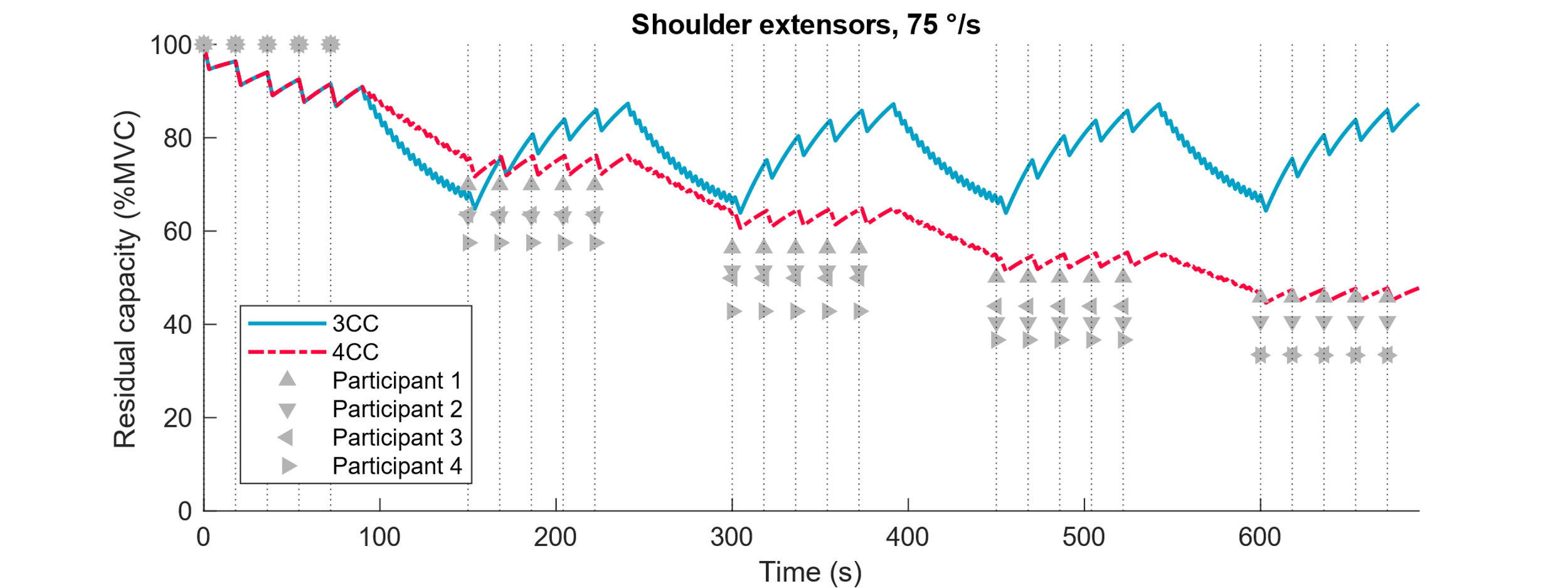


(e)

Figure S1: 3CCr (blue) and 4CCr (red) predictions of fatigue for the shoulder extensors compared to experimental data from four participants (grey triangles). Vertical dotted lines mark sample times.


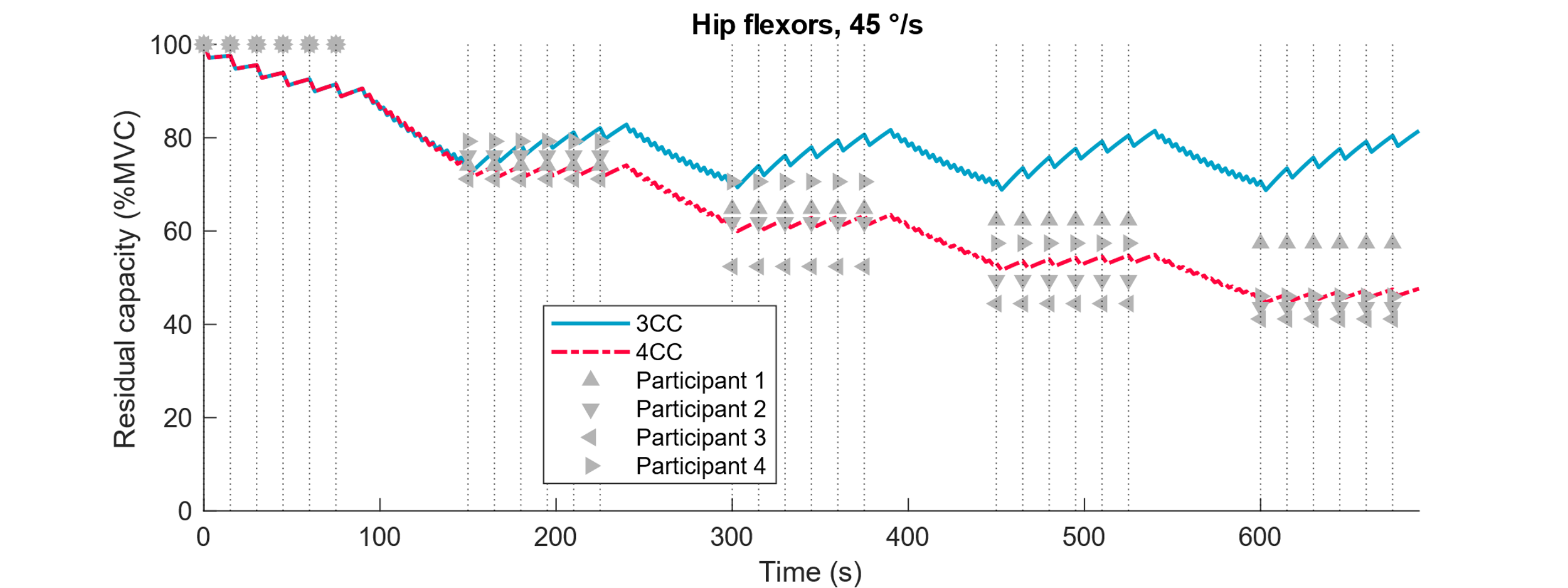


(a)


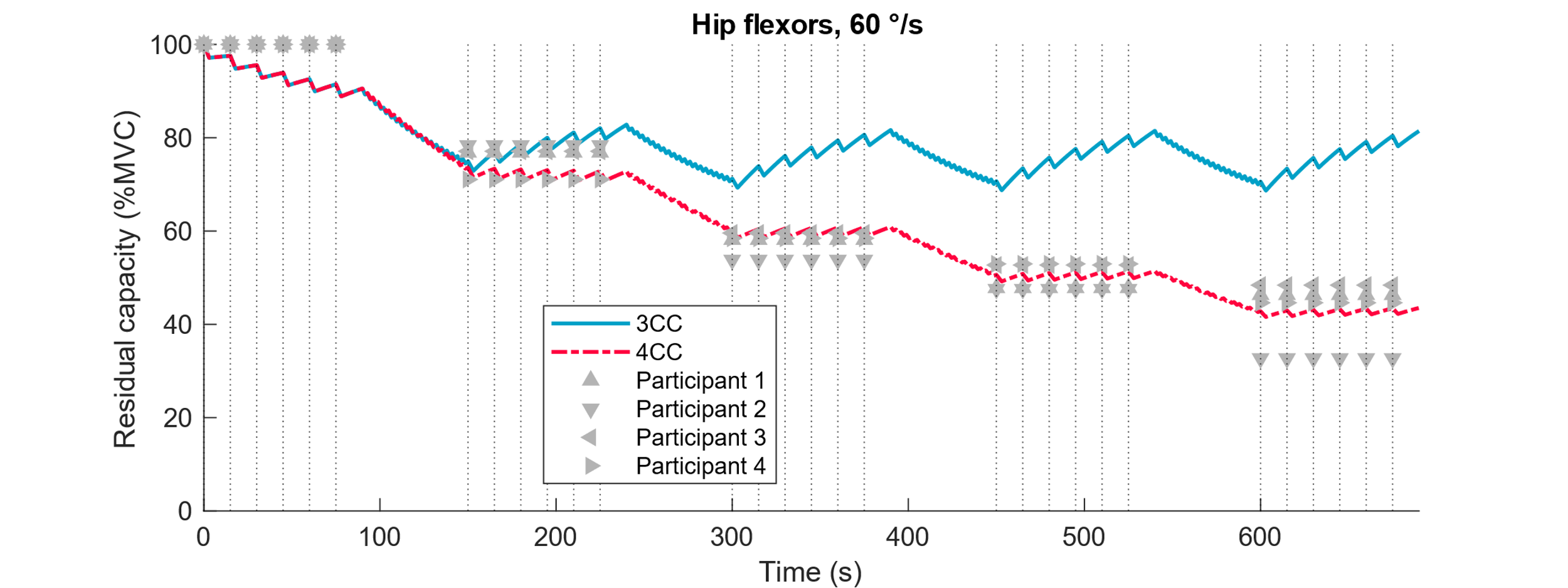


(b)


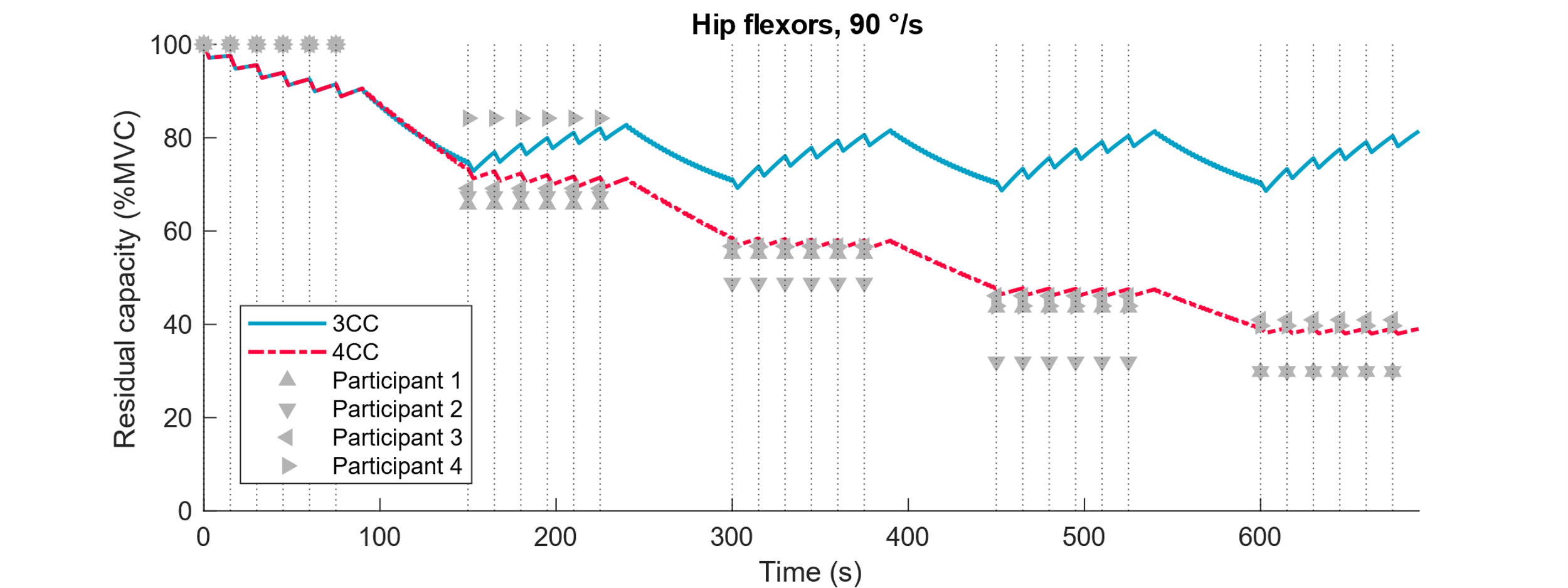


(c)


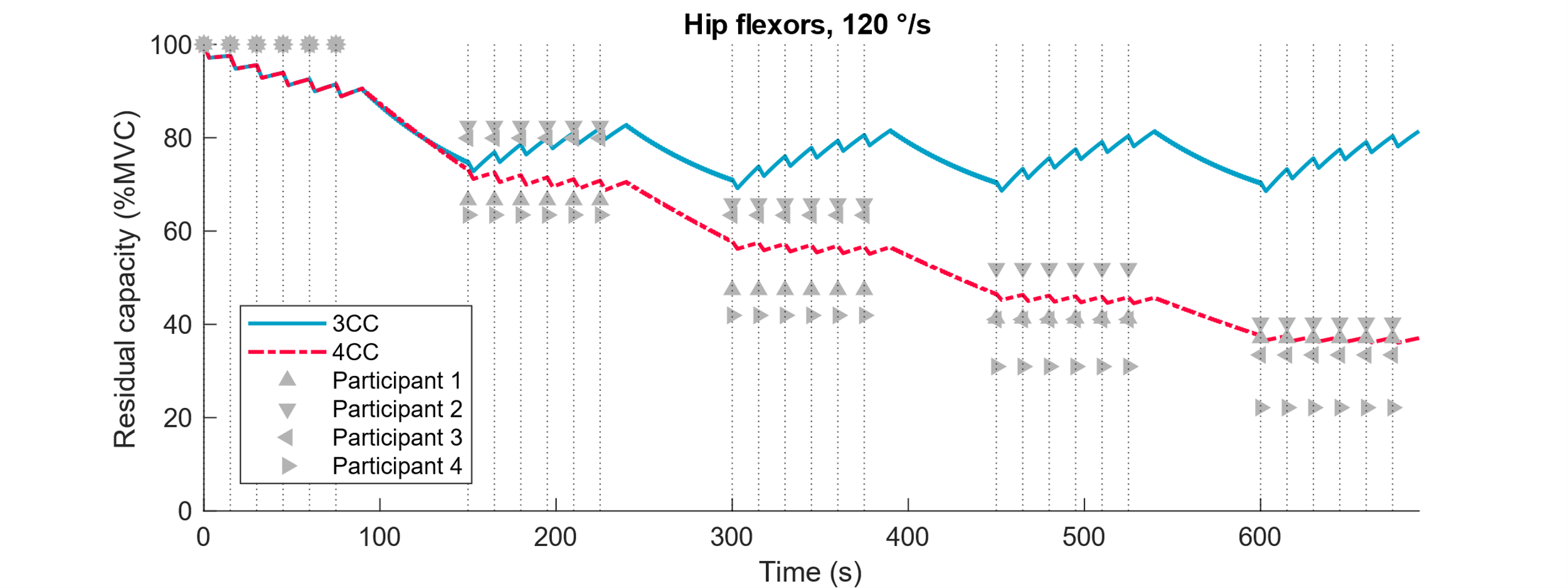


(d)


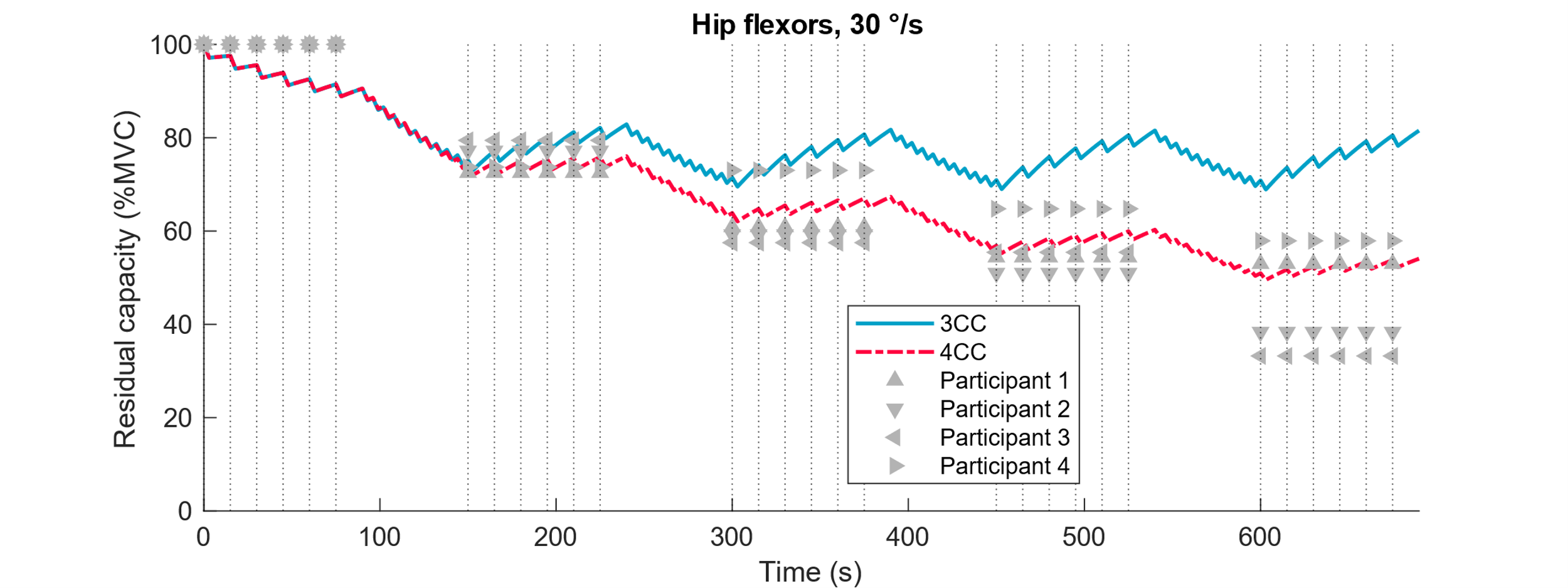


(e)

Figure S2: 3CCr (blue) and 4CCr (red) predictions of fatigue for the hip flexors compared to experimental data from four participants (grey triangles). Vertical dotted lines mark sample times.


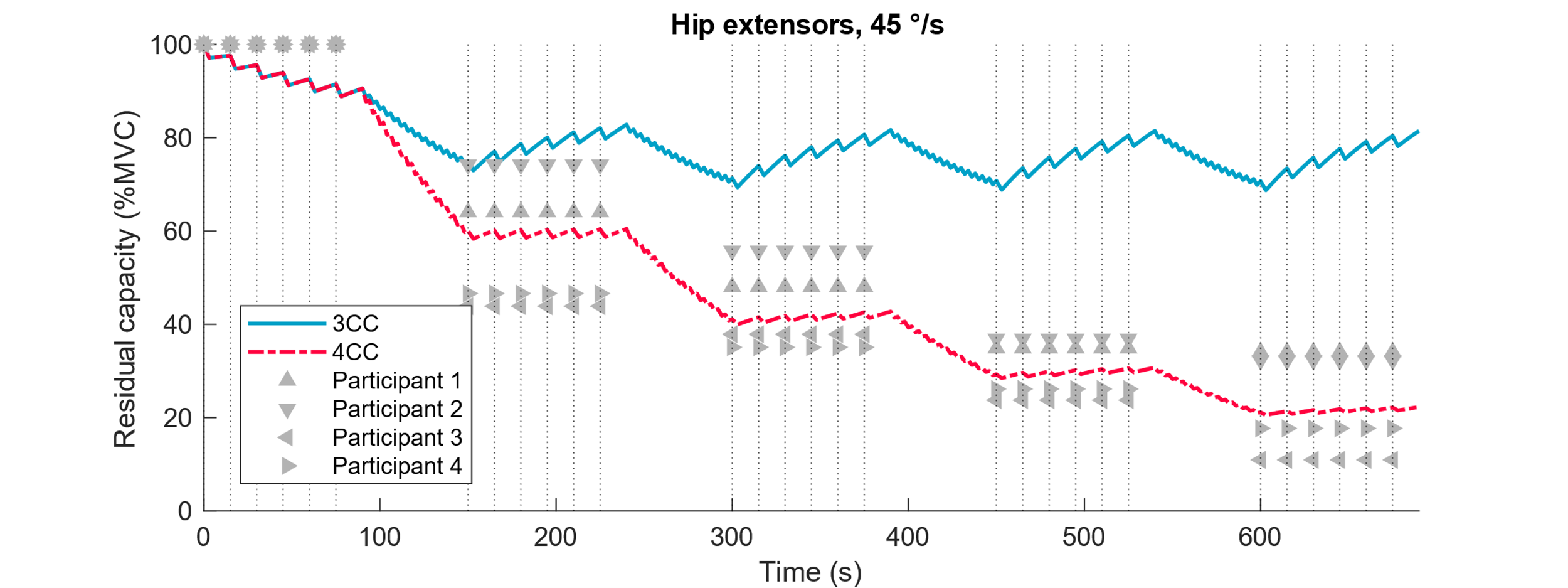


(a)


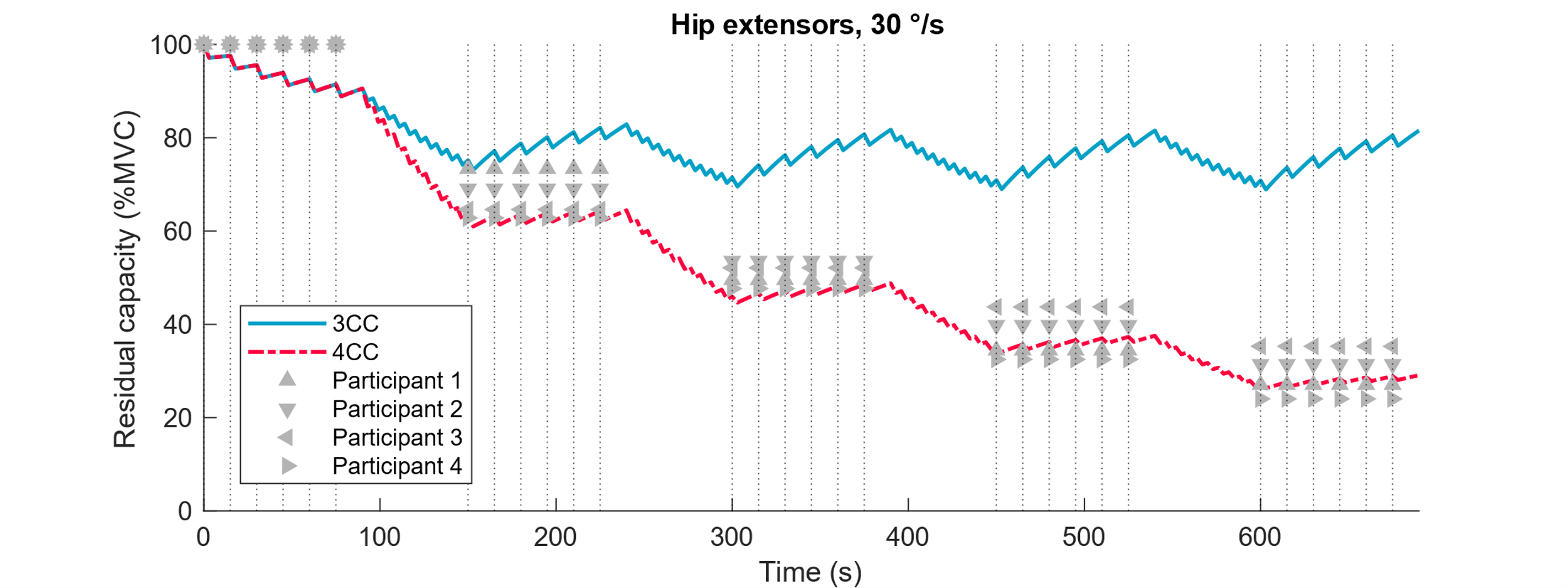


(b)


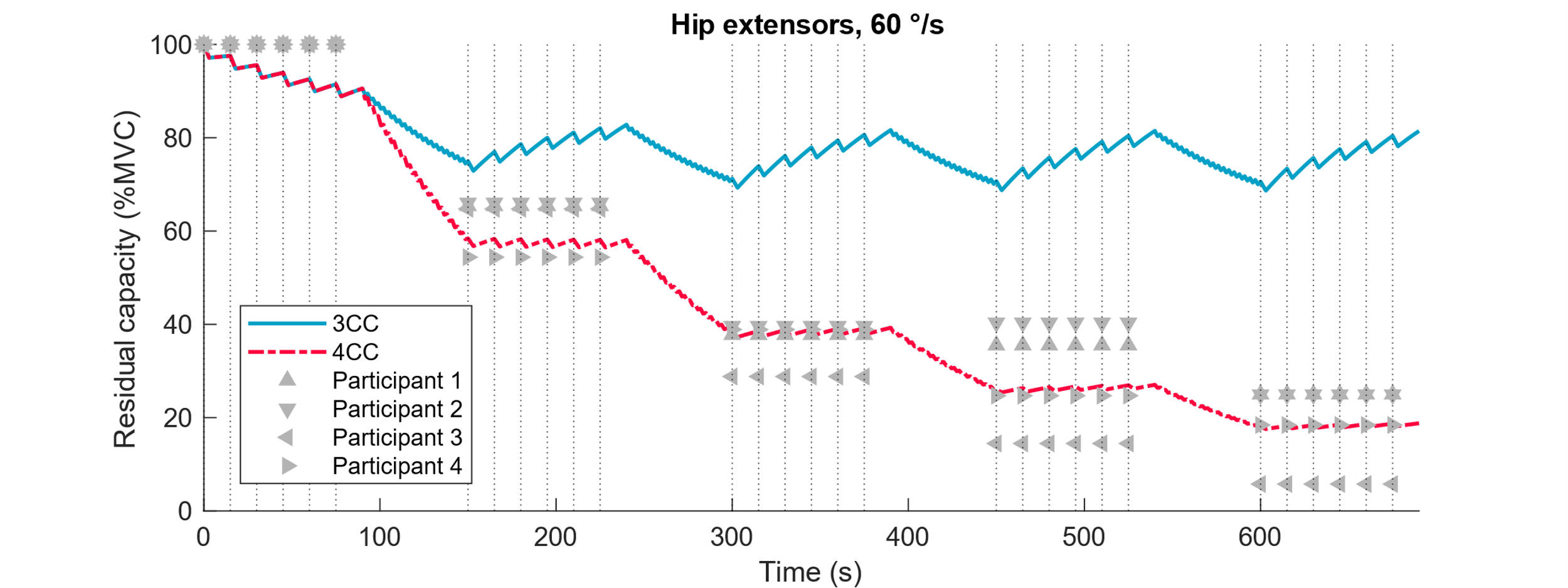


(c)


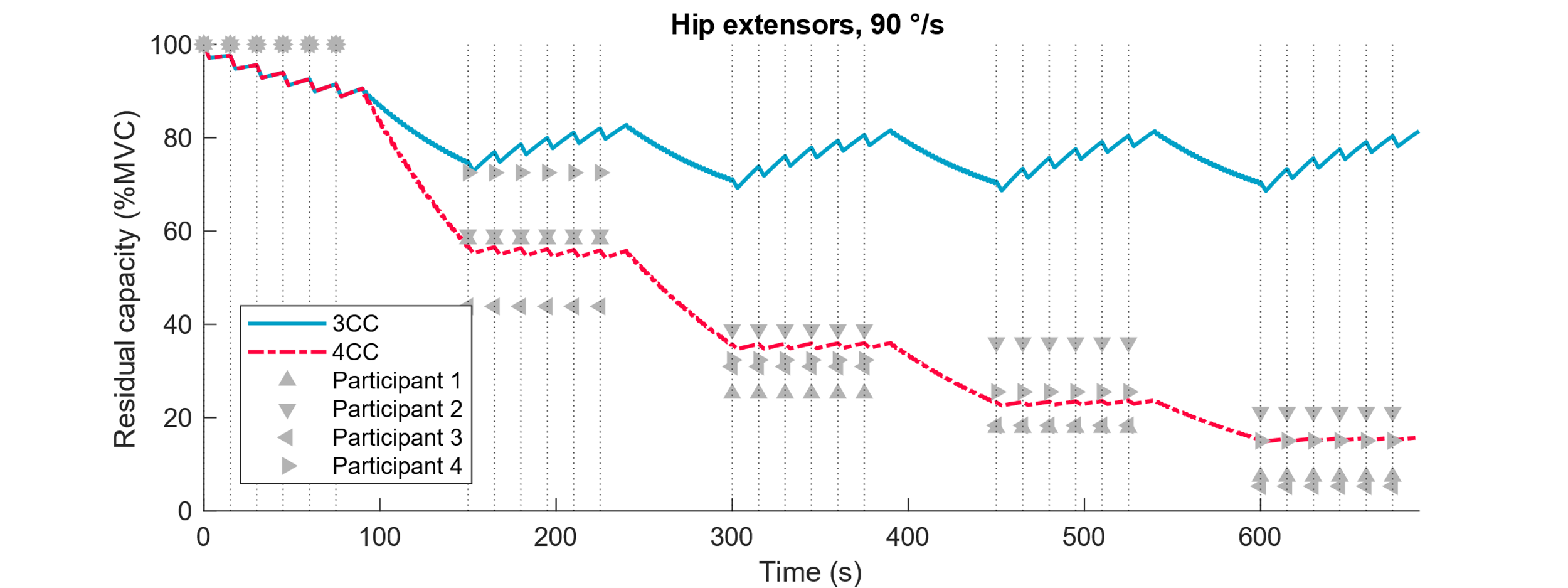


(d)


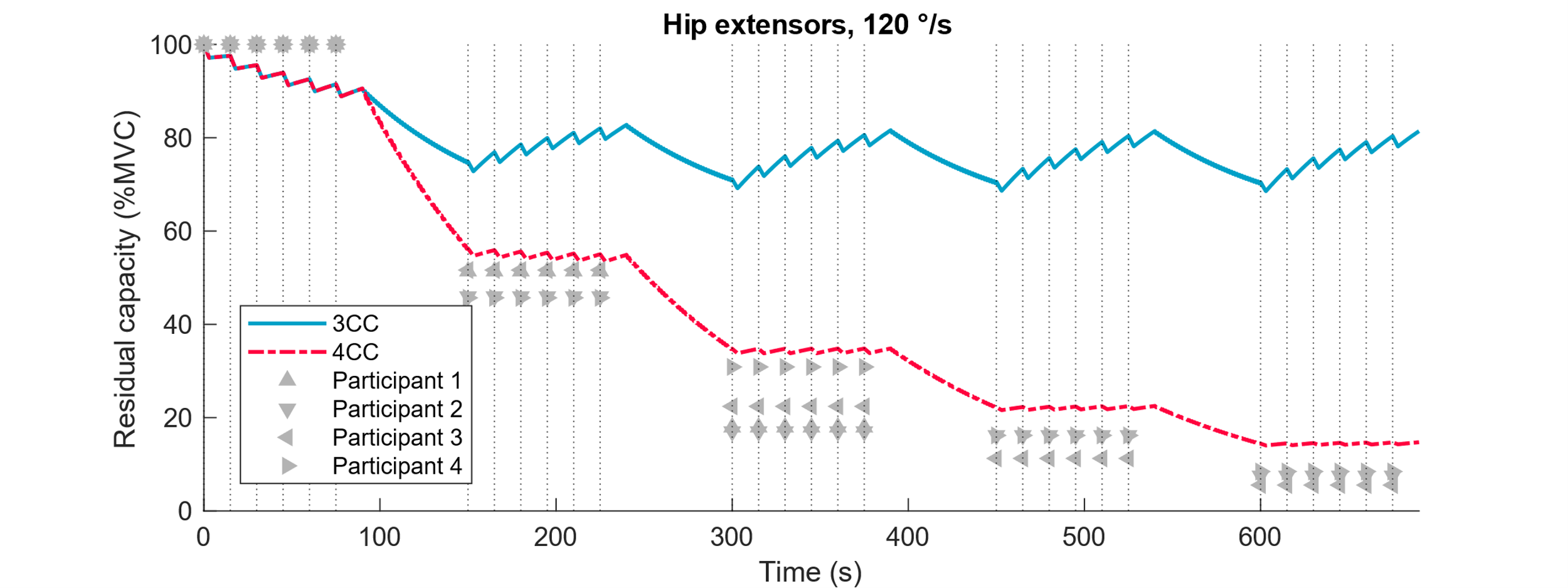


(e)

Figure S3: 3CCr (blue) and 4CCr (red) predictions of fatigue for the hip extensors compared to experimental data from four participants (grey triangles). Vertical dotted lines mark sample times.


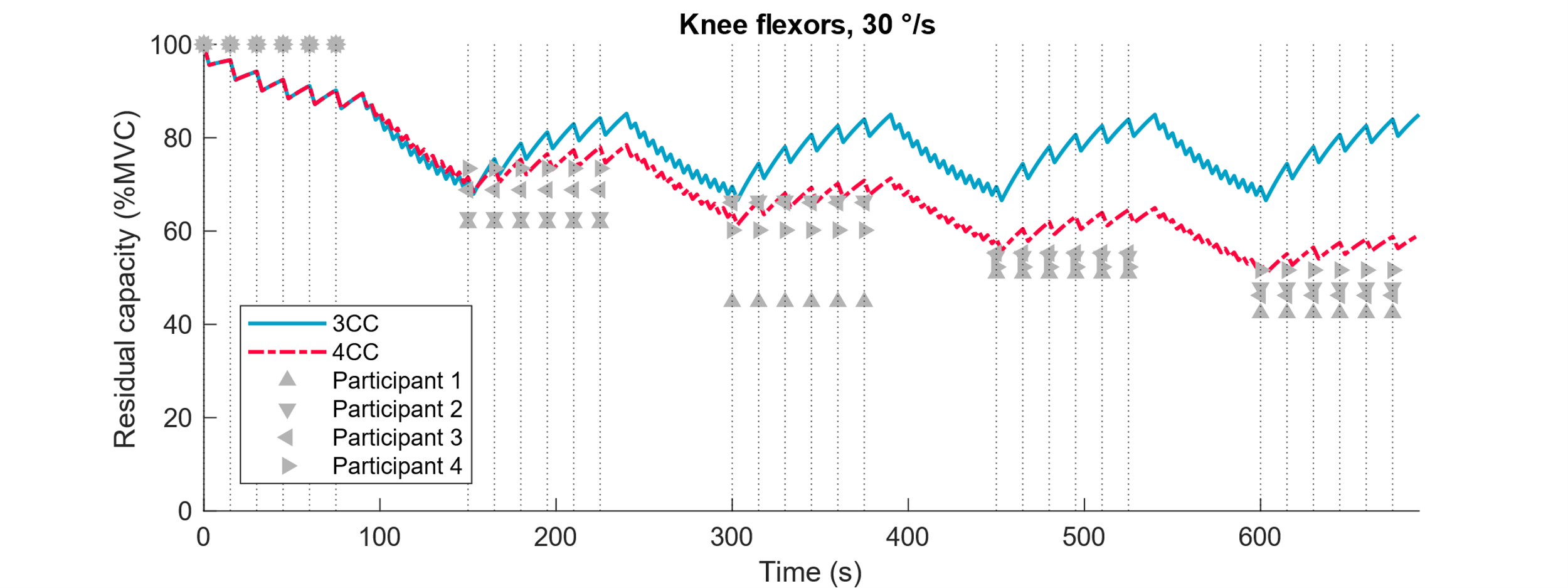


(a)


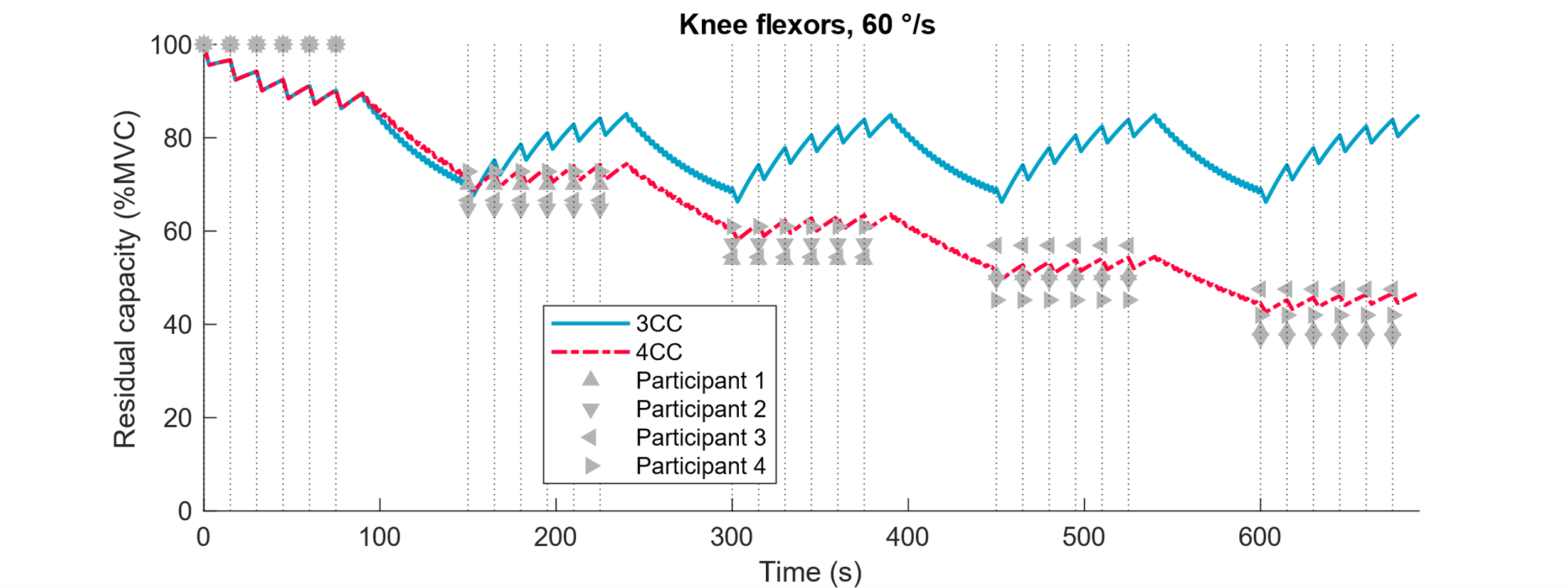


(b)


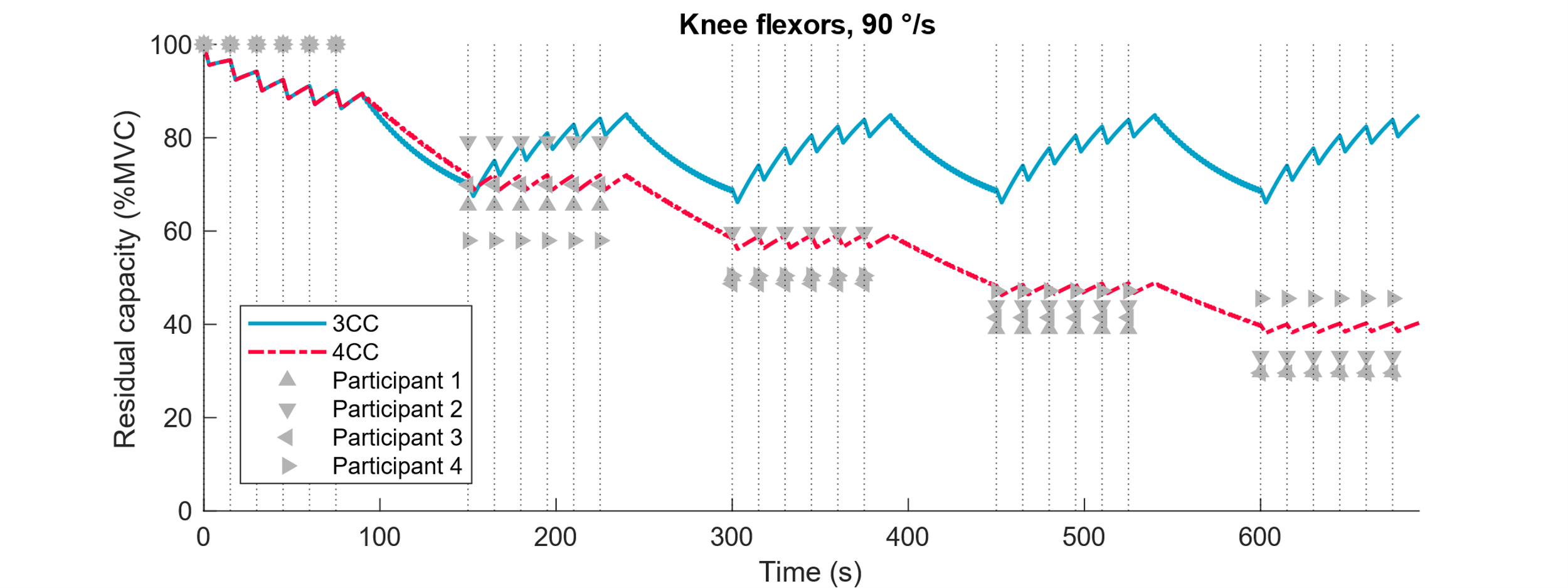


(c)


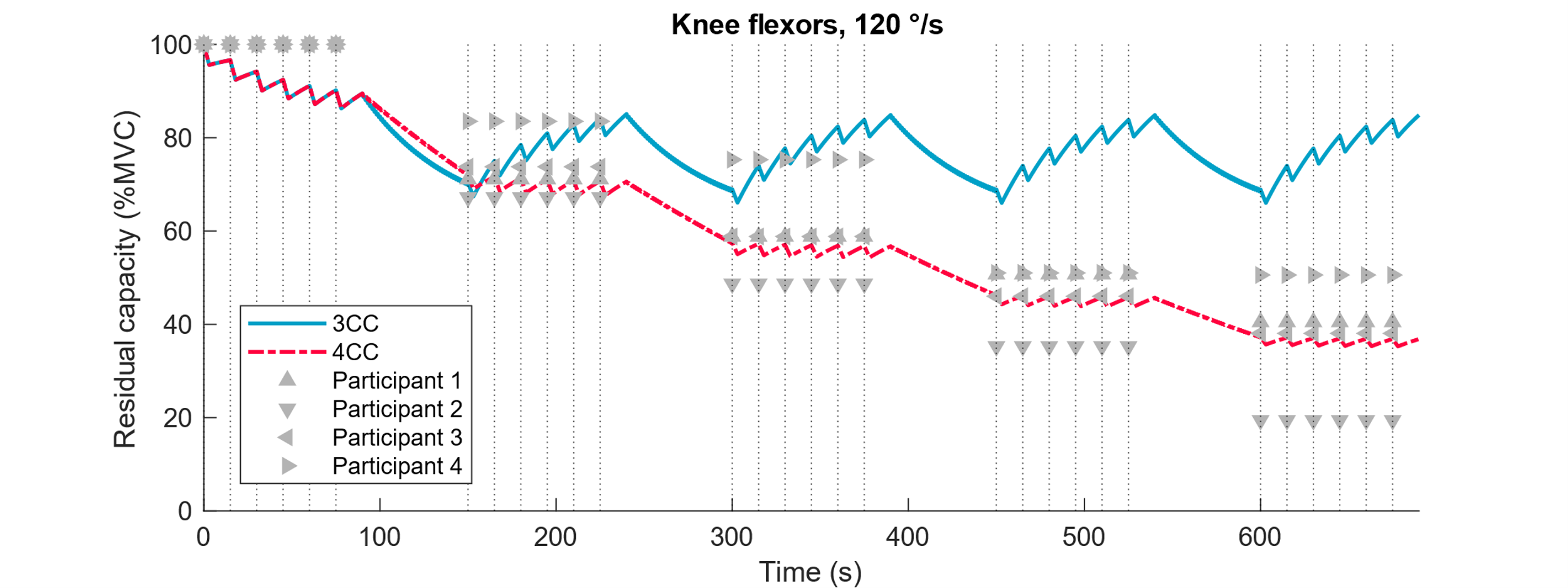


(d)


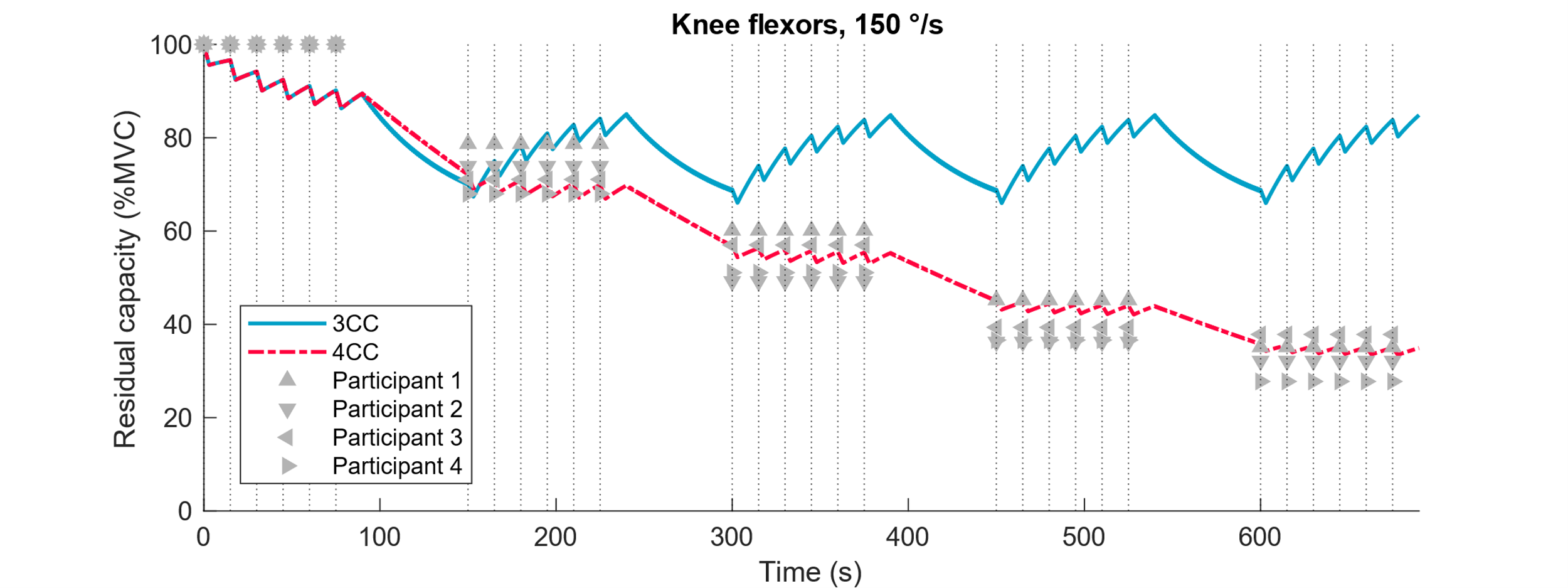


(e)

Figure S4: 3CCr (blue) and 4CCr (red) predictions of fatigue for the knee flexors compared to experimental data from four participants (grey triangles). Vertical dotted lines mark sample times.


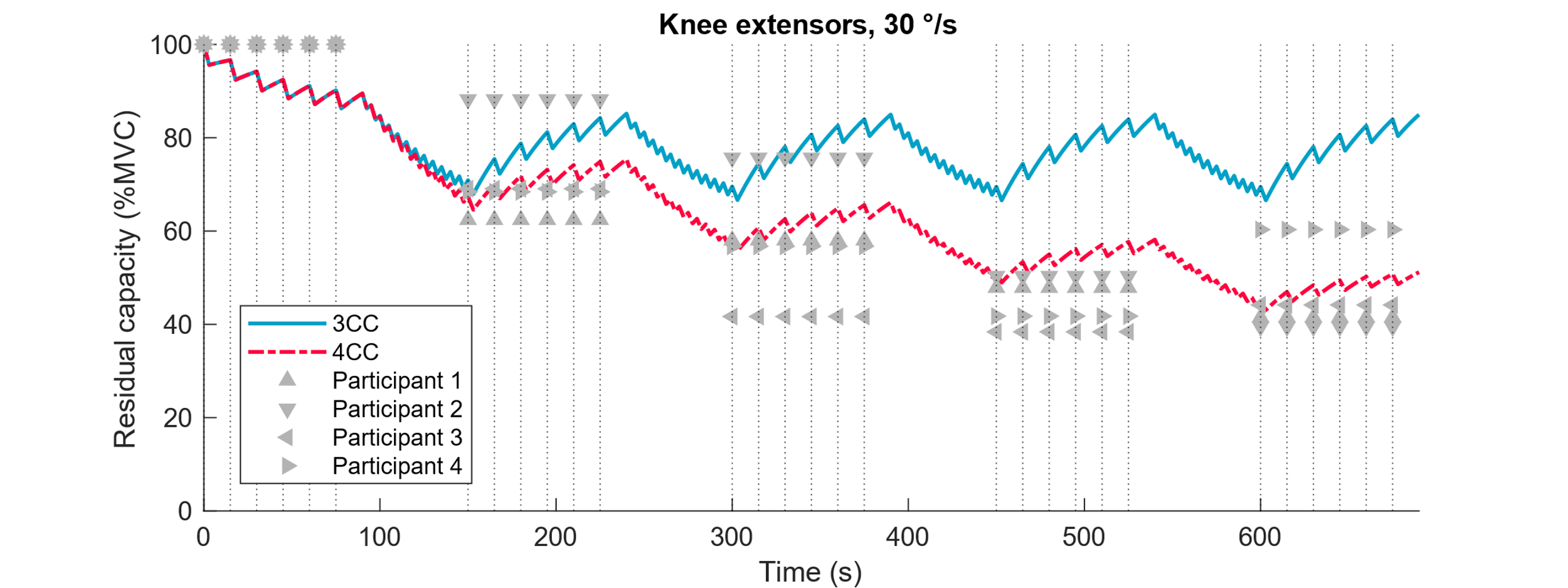


(a)


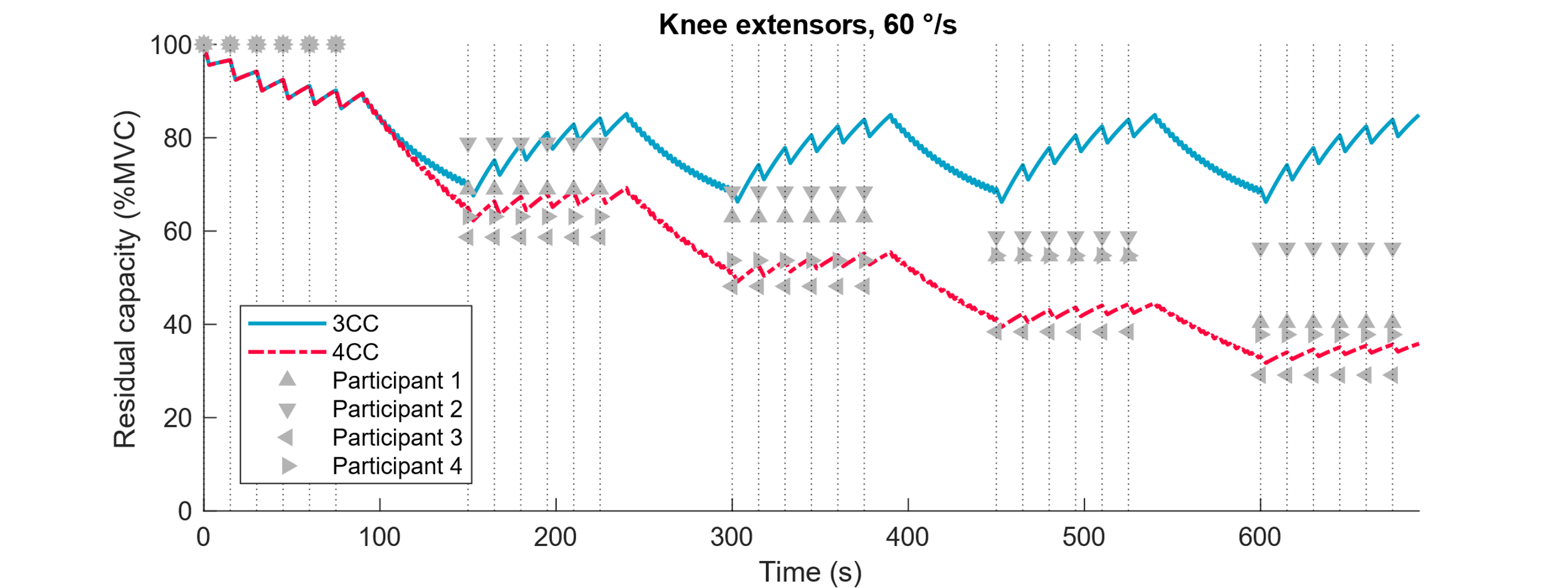


(b)


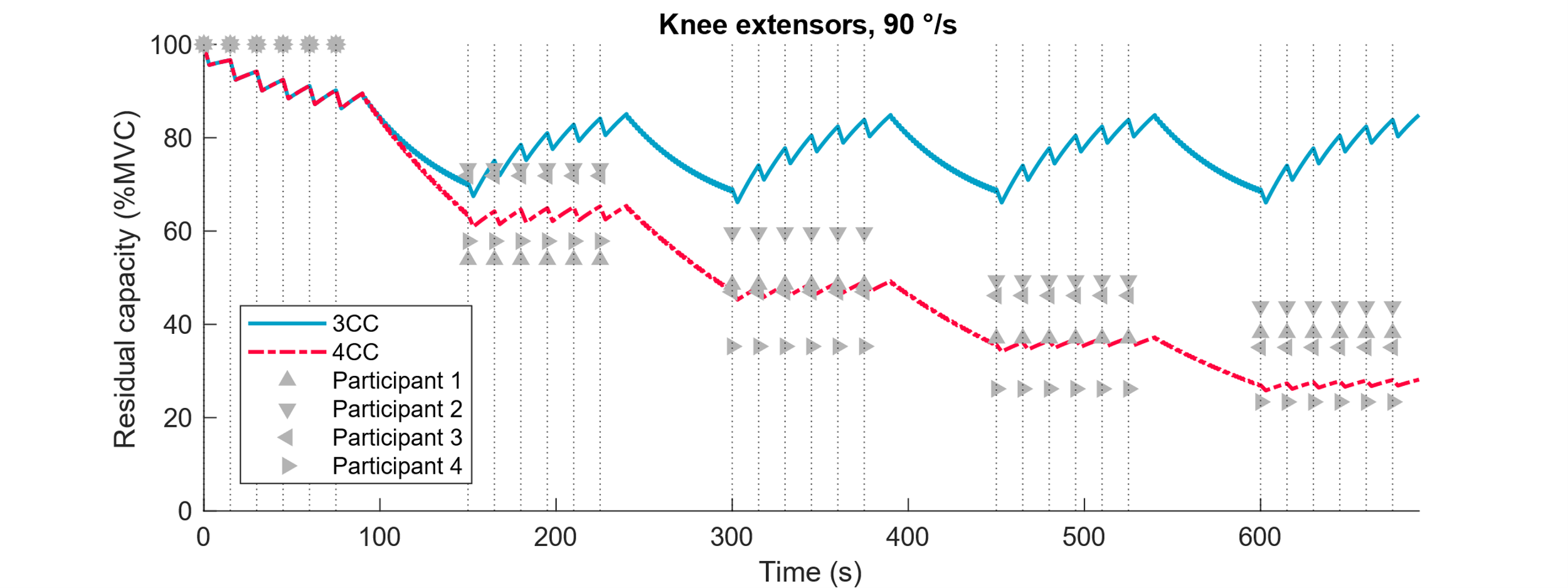


(c)


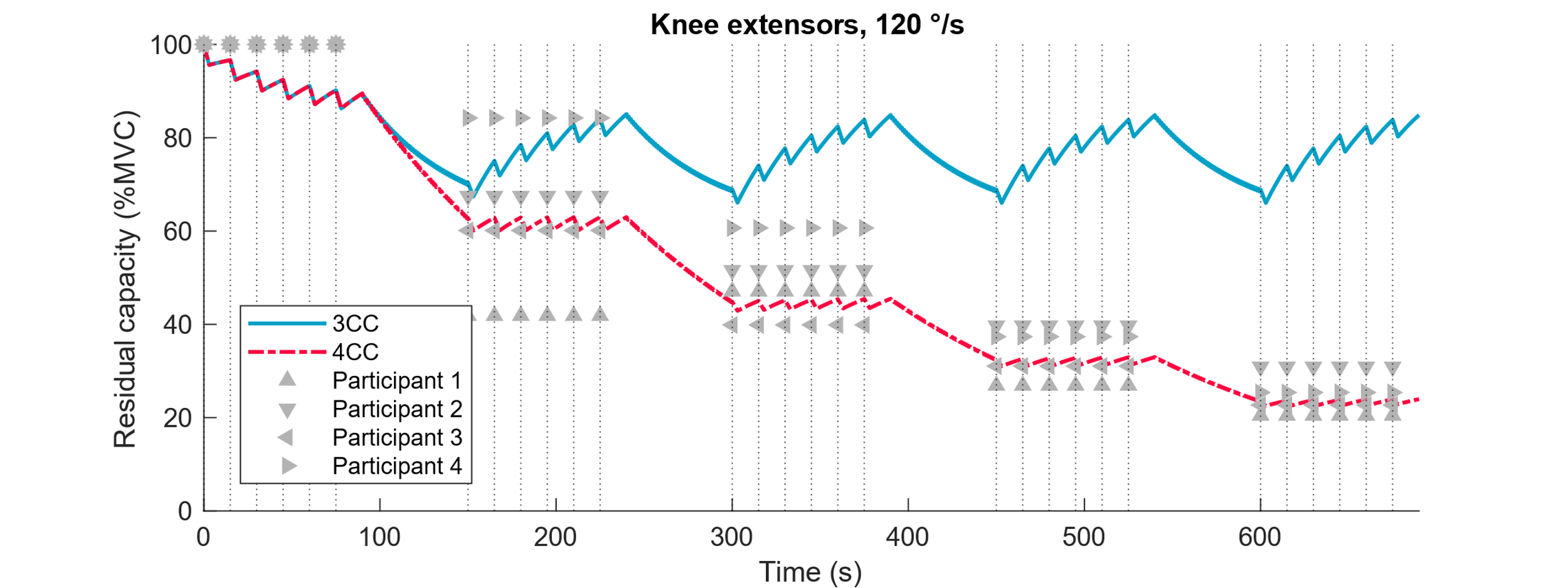


(d)


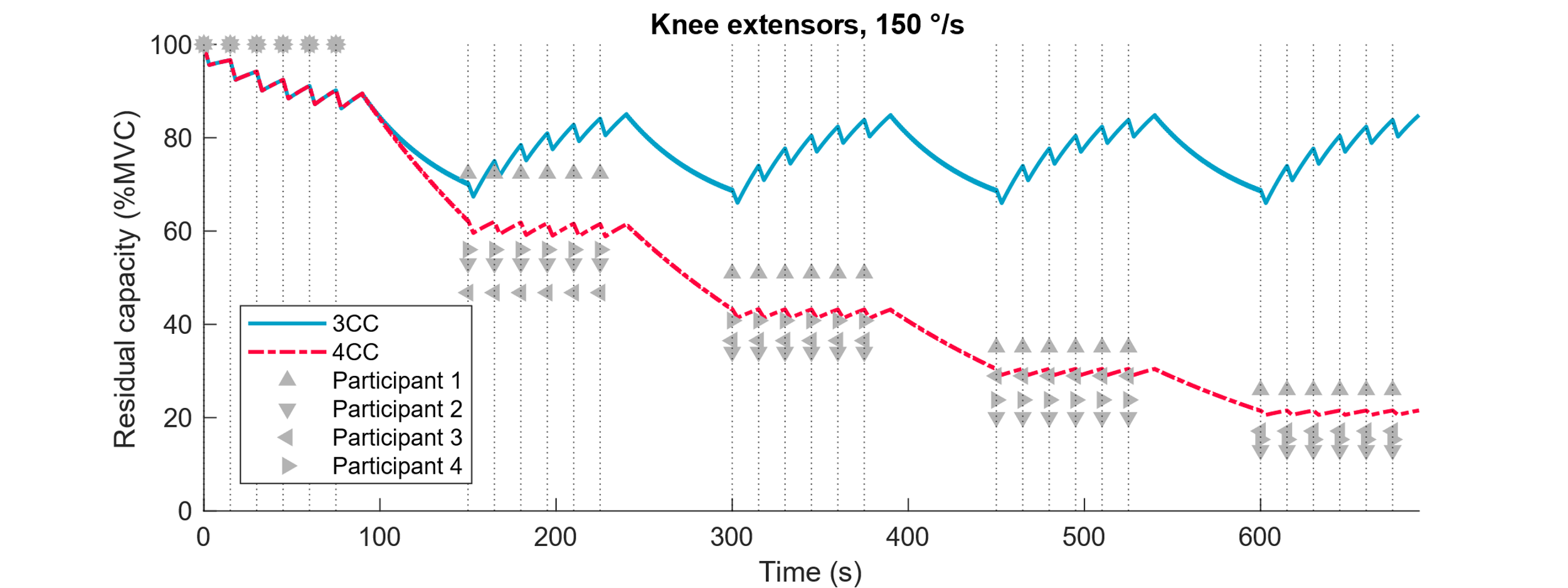


(e)

Figure S5: 3CCr (blue) and 4CCr (red) predictions of fatigue for the knee extensors compared to experimental data from four participants (grey triangles). Vertical dotted lines mark sample times.

Table S1. Pearson's correlation coefficients of 3CCr and 4CCr predictions for shoulder extensors to experimental data from four participants.

| **Joint velocity (**°/s**)** | **20** | | **30** | | **45** | | **60** | | **75** | |
| --- | --- | --- | --- | --- | --- | --- | --- | --- | --- | --- |
| **Participant** | **3CC** | **4CC** | **3CC** | **4CC** | **3CC** | **4CC** | **3CC** | **4CC** | **3CC** | **4CC** |
| 1 | 0.68 | 0.95 | 0.68 | 0.97 | 0.70 | 0.96 | 0.68 | 0.97 | 0.68 | 0.98 |
| 2 | 0.73 | 0.92 | 0.65 | 0.97 | 0.70 | 0.96 | 0.70 | 0.96 | 0.69 | 0.97 |
| 3 | 0.73 | 0.87 | 0.73 | 0.91 | 0.71 | 0.95 | 0.65 | 0.99 | 0.67 | 0.98 |
| 4 | 0.69 | 0.95 | 0.63 | 0.96 | 0.72 | 0.92 | 0.72 | 0.93 | 0.70 | 0.96 |

Table S2. Pearson's correlation coefficients of 3CCr and 4CCr predictions for hip flexors to experimental data from four participants.

| **Joint velocity (**°/s**)** | **30** | | **45** | | **60** | | **90** | | **120** | |
| --- | --- | --- | --- | --- | --- | --- | --- | --- | --- | --- |
| **Participant** | **3CC** | **4CC** | **3CC** | **4CC** | **3CC** | **4CC** | **3CC** | **4CC** | **3CC** | **4CC** |
| 1 | 0.89 | 0.98 | 0.90 | 0.97 | 0.84 | 0.99 | 0.84 | 0.99 | 0.88 | 0.98 |
| 2 | 0.81 | 0.99 | 0.83 | 1.00 | 0.79 | 0.99 | 0.84 | 0.99 | 0.76 | 0.99 |
| 3 | 0.77 | 0.97 | 0.86 | 0.99 | 0.85 | 0.99 | 0.87 | 0.99 | 0.76 | 0.99 |
| 4 | 0.87 | 0.97 | 0.79 | 0.99 | 0.87 | 0.99 | 0.77 | 0.98 | 0.85 | 0.99 |

Table S3. Pearson's correlation coefficients of 3CCr and 4CCr predictions for hip extensors to experimental data from four participants.

| **Joint velocity (**°/s**)** | **30** | | **45** | | **60** | | **90** | | **120** | |
| --- | --- | --- | --- | --- | --- | --- | --- | --- | --- | --- |
| **Participant** | **3CC** | **4CC** | **3CC** | **4CC** | **3CC** | **4CC** | **3CC** | **4CC** | **3CC** | **4CC** |
| 1 | 0.81 | 0.99 | 0.87 | 0.99 | 0.86 | 0.99 | 0.85 | 0.99 | 0.85 | 1.00 |
| 2 | 0.84 | 1.00 | 0.81 | 0.99 | 0.85 | 0.99 | 0.87 | 0.99 | 0.89 | 0.99 |
| 3 | 0.88 | 0.99 | 0.88 | 0.98 | 0.82 | 0.99 | 0.88 | 0.99 | 0.87 | 1.00 |
| 4 | 0.85 | 1.00 | 0.90 | 0.98 | 0.88 | 1.00 | 0.80 | 0.98 | 0.89 | 0.99 |

Table S4. Pearson's correlation coefficients of 3CCr and 4CCr predictions for knee flexors to experimental data from four participants.

| **Joint velocity (**°/s**)** | **30** | | **60** | | **90** | | **120** | | **150** | |
| --- | --- | --- | --- | --- | --- | --- | --- | --- | --- | --- |
| **Participant** | **3CC** | **4CC** | **3CC** | **4CC** | **3CC** | **4CC** | **3CC** | **4CC** | **3CC** | **4CC** |
| 1 | 0.77 | 0.94 | 0.72 | 0.99 | 0.72 | 0.99 | 0.72 | 0.99 | 0.64 | 0.99 |
| 2 | 0.76 | 0.95 | 0.74 | 0.98 | 0.63 | 0.99 | 0.68 | 1.00 | 0.68 | 0.99 |
| 3 | 0.73 | 0.97 | 0.77 | 0.95 | 0.70 | 0.99 | 0.69 | 1.00 | 0.70 | 0.99 |
| 4 | 0.74 | 0.97 | 0.70 | 0.99 | 0.79 | 0.92 | 0.60 | 0.97 | 0.70 | 1.00 |

Table S5: Pearson's correlation coefficients of 3CCr and 4CCr predictions for knee extensors to experimental data from four participants.

| **Joint velocity (**°/s**)** | **30** | | **60** | | **90** | | **120** | | **150** | |
| --- | --- | --- | --- | --- | --- | --- | --- | --- | --- | --- |
| **Participant** | **3CC** | **4CC** | **3CC** | **4CC** | **3CC** | **4CC** | **3CC** | **4CC** | **3CC** | **4CC** |
| 1 | 0.76 | 0.97 | 0.71 | 0.98 | 0.78 | 0.95 | 0.76 | 0.94 | 0.67 | 1.00 |
| 2 | 0.54 | 0.92 | 0.71 | 0.99 | 0.71 | 1.00 | 0.71 | 1.00 | 0.74 | 0.99 |
| 3 | 0.73 | 0.93 | 0.75 | 0.99 | 0.71 | 0.98 | 0.73 | 0.99 | 0.77 | 0.97 |
| 4 | 0.73 | 0.88 | 0.75 | 0.96 | 0.74 | 0.98 | 0.58 | 0.97 | 0.72 | 0.99 |
